# Usefulness of the combination of a single dose of neostigmine-atropine plus antivenom on the experimental treatment of the envenomation caused by *Micrurus* venoms

**DOI:** 10.48327/mtsi.v6i3.2026.871

**Published:** 2026-07-02

**Authors:** Adolfo Rafael DE ROODT, Daniel DOZORETZ, Fernando Andrés MORÓN GOÑI, Marcela Alejandra DESIO, Carlos Fabián DAMÍN

**Affiliations:** 1 Primera Catedra de Toxicologia, Carrera de Medicina, Facultad de Ciencias Medicas, Universidad de Buenos Aires. Paraguay 2155, CP 1121, CABA, Argentina.; 2 Area Investigacion y Desarrollo – Venenos. Instituto Nacional de Produccion de Biologicos, Administracion Nacional de Laboratorios e Institutos de Salud “Dr. Carlos G. Malbran”. Av. Velez Sarsfield 563, CP1282, CABA, Argentina.

**Keywords:** *Micrurus*, Coral snake, Venom, Envenomation, Neostigmine, Acetylcholinesterase Inhibitor, Atropine, Treatment, Antivenom, α-neurotoxins, β-neurotoxins, Three-Finger toxins, Phospholipases, Neurotoxicity, *Micrurus*, Serpent corail, Venin, Envenimation, Néostigmine, Inhibiteur de l’acétylcholinestérase, Atropine, Traitement, Antivenin, α-neurotoxines, β-neurotoxines, Toxines à trois doigts, Phospholipases, Neurotoxicité, *Micrurus*, Serpiente de coral, Veneno, Envenenamiento, Neostigmina, Inhibidor de acetilcolinesterasa, Atropina, Tratamiento, Antiveneno, α-neurotoxinas, β-neurotoxinas, Toxinas de tres dedos, Fosfolipasas, Neurotoxicidad

## Abstract

**Introduction:**

Envenomation by *Micrurus* snakes is relatively infrequent, but constitutes a persistent public health concern throughout the Americas, due to its potentially severe clinical consequences. The venom of these snakes, like that of other elapids, primarily contains postsynaptic toxins (α-neurotoxins, with a three-finger toxin (3-FTx) structure) and some species also produce presynaptic toxins (β-neurotoxins, with a phospholipase A_2_ (PLA_2_) structure). Given this composition, the potential therapeutic value of combining an acetylcholinesterase inhibitor, neostigmine (N) plus atropine (A) with an anti-*Micrurus* antivenom (AV) was investigated. The efficacy of this combination therapy was evaluated in comparison to AV alone, using experimental envenomation models involving *Micrurus* venoms with different proportions of 3-FTxs and PLA_2_s.

**Methods:**

Groups of mice were challenged with 1.2 minimal mortal doses of *M. pyrrhocryptus* (Mp), *M. altirostris* (Ma), *M. surinamensis* (Ms), *M. nigrocinctus* (Mn), or *M. fulvius* (Mf) venom. After 15 minutes, the animals received a single rescue treatment with either N+A, various doses of anti-*Micrurus* AV alone, or a combination of AV plus N+A. Survival was assessed at 24 hours. For the venom control groups and those treated with N+A alone, time to death was also recorded. The median effective dose of AV alone, or with the addition of N+A, was estimated for Mp and Ma venoms.

**Results:**

Treatment with the N+A combination alone did not provide effective protection. However, N+A slightly delayed the time to death in animals envenomed with Ms, Ma, and Mp venoms (p < 0.05). The combination of N+A with AV significantly improved survival compared to AV alone in mice challenged with Ma and Mp venoms (p < 0.05). In contrast, higher AV doses were required to achieve similar effects in animals envenomed with Ms venom, while Mn and Mf venoms required the highest AV doses to show some level of protection. The estimated median effective dose of AV in combination with N+A was more potent, enhancing AV potency by 1.6-fold for Mp and up to 3.4-fold for Ma venom.

**Conclusion:**

The survival of mice treated with AV plus N+A was higher in envenomations caused by Ma, Ms, and Mp venoms, which are characterized by prominent α-neurotoxic activity and a lower proportion of PLA_2_s. The effect was less evident and only apparent at the highest AV doses for Mn and Mf venoms, which contain higher levels of PLA_2_s and exhibit strong experimental myotoxic capacity. The results show that the combined use of N+A and AV enhances the effectiveness of treatment in *Micrurus* envenomations, especially those involving predominantly postsynaptic neurotoxins, by reducing the AV dose required for recovery.

## RÉSUMÉ

**Introduction:**

Les envenimations causées par les serpents du genre *Micrurus* sont relativement rares, mais constituent un problème de santé publique persistant dans l’ensemble des Amériques en raison de leurs conséquences cliniques potentiellement graves. Le venin de ces serpents, à l’instar de celui d’autres élapidés, contient principalement des toxines postsynaptiques (α-neurotoxines, de structure « trois doigts » (3-FTx)) et certaines espèces produisent également des toxines présynaptiques (β-neurotoxines, de structure phospholipase A_2_ (PLA_2_)). Compte tenu de cette composition, la valeur thérapeutique potentielle de l’association d’un inhibiteur de l’acétylcholinestérase, la néostigmine (N), et de l’atropine (A) avec un antivenin anti-*Micrurus* (AV) a été étudiée. L’efficacité de cette thérapie combinée a été évaluée par rapport à l’AV seul, à l’aide de modèles expérimentaux d’envenimation impliquant des venins de *Micrurus* contenant différentes proportions de 3-FTx et de PLA_2_.

**Méthode:**

Des groupes de souris ont été exposés à 1,2 dose létale minimale de venin de *M. pyrrhocryptus* (Mp), *M. altirostris* (Ma), *M. surinamensis* (Ms), *M. nigrocinctus* (Mn) ou *M. fulviu*s (Mf). Au bout de 15 minutes, les animaux ont reçu un traitement de secours unique comprenant soit N+A, soit différentes doses d’AV anti-*Micrurus* seul, soit une combinaison d’AV et de N+A. La survie a été évaluée à 24 heures. Pour les groupes témoins exposés au venin et ceux traités par N+A seul, le délai avant la mort a également été enregistré. La dose efficace médiane de l’AV seul, ou en association avec N+A, a été estimée pour les venins de Mp et de Ma.

**Résultats:**

Le traitement par la combinaison N+A seule n’a pas fourni de protection efficace. Cependant, N+A a légèrement retardé le délai avant la mort chez les animaux envenimés par les venins de Ms, Ma et Mp (p < 0,05). La combinaison de N+A et d’AV a significativement amélioré la survie par rapport à l’AV seul chez les souris exposées aux venins de Ma et Mp (p < 0,05).

En revanche, des doses plus élevées d’AV ont été nécessaires pour obtenir des effets similaires chez les animaux envenimés par le venin de Ms, tandis que les venins de Mn et de Mf ont nécessité les doses d’AV les plus élevées pour montrer un certain niveau de protection. La dose efficace médiane estimée d’AV en association avec N+A était plus puissante, augmentant la puissance de l’AV de 1,6 fois pour le venin de Mp et jusqu’à 3,4 fois pour le venin de Ma.

**Conclusion:**

Le taux de survie des souris traitées par AV plus N+A était plus élevé dans les cas d’envenimations causées par les venins de Ma, Ms et Mp, qui se caractérisent par une activité α-neurotoxique marquée et une proportion plus faible de PLA_2_. L’effet était moins évident et n’apparaissait qu’aux doses les plus élevées d’AV pour les venins de Mn et Mf, qui contiennent des niveaux plus élevés de PLA_2_ et présentent une forte capacité myotoxique expérimentale. Les résultats montrent que l’utilisation combinée de N+A et d’AV améliore l’efficacité du traitement dans les envenimations par *Micrurus*, en particulier celles impliquant principalement des neurotoxines postsynaptiques, en réduisant la dose d’AV nécessaire à la guérison.

## Resumen

**Introducción:**

Los envenenamientos por *Micrurus* constituyen un problema sanitario de baja frecuencia, pero constante en América, siendo en todos los casos potencialmente graves. El veneno de estas serpientes como el de otros elápidos posee mayoritariamente toxinas postsinápticas (α-neurotoxinas, con estructura de 3-FTx) y algunas, también poseen toxinas presinápticas (β-neurotoxinas, con estructura de fosfolipasas (PLA_2_)). Dado que el uso de inhibidores de la acetil colinesterasa se mostró util en algunos casos de envenenamiento por venenos con 3-FTxs, se evaluó la utilidad de la neostigmina (N) + atropina (A) y su combinación con la aplicación de antiveneno (AV) anti-*Micrurus*, respecto al uso de AV solamente, en el envenenamiento experimental por el veneno de *Micrurus* con diferentes proporciones de 3-FTxs y PLA_2_s.

**Métodos:**

Grupos de ratones fueron desafiados con 1.2 dosis mínimas mortales de veneno de *M. pyrrhocryptus* (Mp), *M. altirostris* (Ma), *M. surinamensis* (Ms), *M. nigrocinctus* (Mn) o *M. fulviu*s (Mf). A los 15 minutos se aplicaron diferentes dosis de AV solo o de AV más N+A y a las 24 horas se registró la sobrevida. En el caso de los controles de veneno y los tratados con N+A solamente, se registró el tiempo de muerte. Además se estimó la dosis efectiva media del AV aplicado solo, o con el agregado de N+A en los envenenamientos por venenos de Mp y Ma.

**Resultados:**

La combinación N+A en una única dosis como único tratamiento, no protegió a los ratones, sin embargo enlenteció levemente el tiempo de muerte causada por los venenos de Ms, Ma y Mp (p < 0.05). La combinación N+A con el AV, mejoró la sobrevida de los animales desafiados respecto al uso del AV solamente, sobre los venenos de Ma y Mp (p <0.05), requiriendo dosis de AV mayores en el caso de Ms y muy altas en los casos de Mn y Mf. La dosis efectiva media del AV en combinación con N+A, mejoró la potencia neutralizante entre 1.6 (Mp) y 3.4 (Ma) veces.

**Conclusión:**

La sobrevida fue mucho más evidente en el caso de los envenenamientos por venenos de Ma, Ms y Mp, venenos con importante actividad α-neurotóxica y menor proporción de PLA_2_, mientras que fue muy baja y solo evidente a las dosis de AV más altas sobre los venenos de Mn y Mf, venenos con mayor proporción de PLA_2_s y alta capacidad miotóxica experimental. Los resultados muestran que la combinación de N+A con el AV mejora el resultado de la terapéutica del envenenamiento por algunas *Micrurus*, disminuyendo la dosis de AV necesarias para la recuperación de los animales desafiados, especialmente en aquellos causados por venenos que causan preponderantemente parálisis por bloqueo postsináptico.

**Figure:**
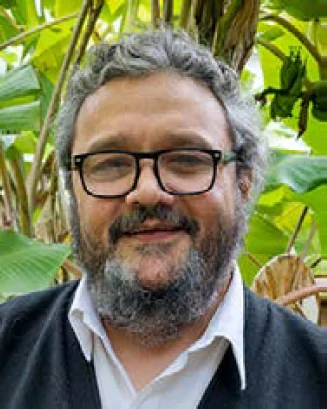

## 1. Introduction

Coral snakes (genera *Micrurus*, *Micruroides* and *Leptomicrurus*) represent the most diversified group of snakes in the Americas. They are the only terrestrial American elapids and inhabit a wide range of terrestrial ecosystems —from sea level to elevations of approximately 3,000 m— from cold and hot desert regions to humid and hot tropical areas, and ranging geographically from northern Patagonia to Mexico and the southern USA [13]. The genus *Micrurus* comprises around 70 species [13,21,58].

The venom of *Micrurus* snakes induces loss of muscle strength and respiratory paralysis of peripheral origin. These neurotoxic effects are primarily due to the action of α-neurotoxins (with a three-finger toxin (3-FTx) structure), which block acetylcholine receptors at the end plate, in the neuromuscular junction, and/or β-neurotoxins (with a phospholipase A_2_ (PLA_2_) structure) that inhibit evoked acetylcholine release by the motor nerve endings at the presynaptic level. In some cases, these effects are further exacerbated by the additive action of myotoxic components on muscle fiber membranes [4,6,10,37,47,67,68,75]. Among the various toxic components of *Micrurus* venoms, the 3-FTx and PLA_2_ neurotoxins are considered the most important [35,42].

The specific treatment for *Micrurus* envenomation is the administration of a corresponding antivenom (AV). Nevertheless, such AVs are scarce, difficult to produce, and currently manufactured by only a limited number of producers [26]. Unlike the broad cross-reactivity and neutralization often observed among AVs targeting viperid venoms in the Americas [5,22,24,61], coral snake AVs generally exhibit limited cross-reactivity [26,27]. This is likely attributable to the qualitative and quantitative differences in the composition of *Micrurus* venoms [42].

The use of acetylcholinesterase inhibitors (AChEIs), such as neostigmine (N), as an adjunct treatment for envenomations due to coral snake bites is suggested by some South American ministries of health [45,46]. Experimental data support the potential usefulness of this type of compounds in reversing *Micrurus* venom induced neurotoxicity [12,20,67]. The AChEIs inhibit acetylcholinesterase activity, thereby increasing acetylcholine levels at the neuromuscular junction, where the excess of acetylcholine can competitively displace the α-neurotoxins from nicotinic receptors [4,37,43]. In elapid envenomation, these drugs are typically administered in combination with atropine (A) to mitigate their muscarinic effects [7,37,43]. Although AChEIs are extensively used in the treatment of envenomation by Asian elapids, whose venoms predominantly contain α-neurotoxins [1,4,29,33,36,37,42,48,56,7 3,74,76], clinical data on their efficacy in *Micrurus* envenomation is not abundant.

In envenomations registered in Brazil, the response to treatment was reported to be favorable when AChEIs were used as an ancillary to AV treatment [10,11]. However, the clinical response to these drugs depends on the presence of α-neurotoxins and the absence or low abundance of β-neurotoxins. Their mechanism of action, as mentioned above, is limited to displacing α-neurotoxins from nicotinic receptors through elevated acetylcholine concentrations at the end-plate [4,37]. *In vitro* studies have demonstrated the effectiveness of AChEIs against experimental envenomation by *M. pyrrhocryptus*, *M. frontalis* and *M. lemniscatus* venoms [12,67], three venoms that contain α-neurotoxins [15, 51,60]. On the other hand, their efficacy was not observed against venoms with a higher content of β-neurotoxins, such as that of *M. corallinus* [19,70]. Beyond the foundational studies of Vital Brazil, experimental *in vivo* research on the use of AChEIs in South American coral snake envenomation is very scarce or lacking entirely [67].

In Argentina eight species of *Micrurus* have been described: *M. pyrrhocryptus, M. altirostris, M. balyocoriphus, M. tricolor, M. frontalis, M. lemniscatus carvalhoi, M. silviae* and *M. corallinus* [26,45]. Among these, *M. pyrrhocryptus* has the widest distribution and is likely responsible for the majority of coral snakebites in the country [26]. Its venom is the primary immunogen used in the production of the AV currently employed in Argentina [23,26]. Only limited data are available on the venoms of species of *Micrurus* described in Argentina [12,25,26,27,28,51], nevertheless the presence of α-neurotoxins in the venoms of these species was described [12,19,28,31,51,60]. This supports the potential role of AChEIs as adjunctive therapies in the treatment of envenomations caused by most of these species.

In this study, we evaluated the efficacy of a single dose of an AChEI, the administration of a South-American *Micrurus* AV alone, and the combination of AChEI plus AV, in experimental murine models of *Micrurus* envenomation. Rescue experiments (experiments in which the antivenom is applied a determinate time after the injection of a lethal dose of venom) were conducted in *M. pyrrhocryptus* (Fig. 1), *M. altirostris* (Fig. 2)*, M. surinamensis* (Fig. 3), *M. nigrocinctus* and *M. fulvius* experimental envenomations.

**Figure 1:**
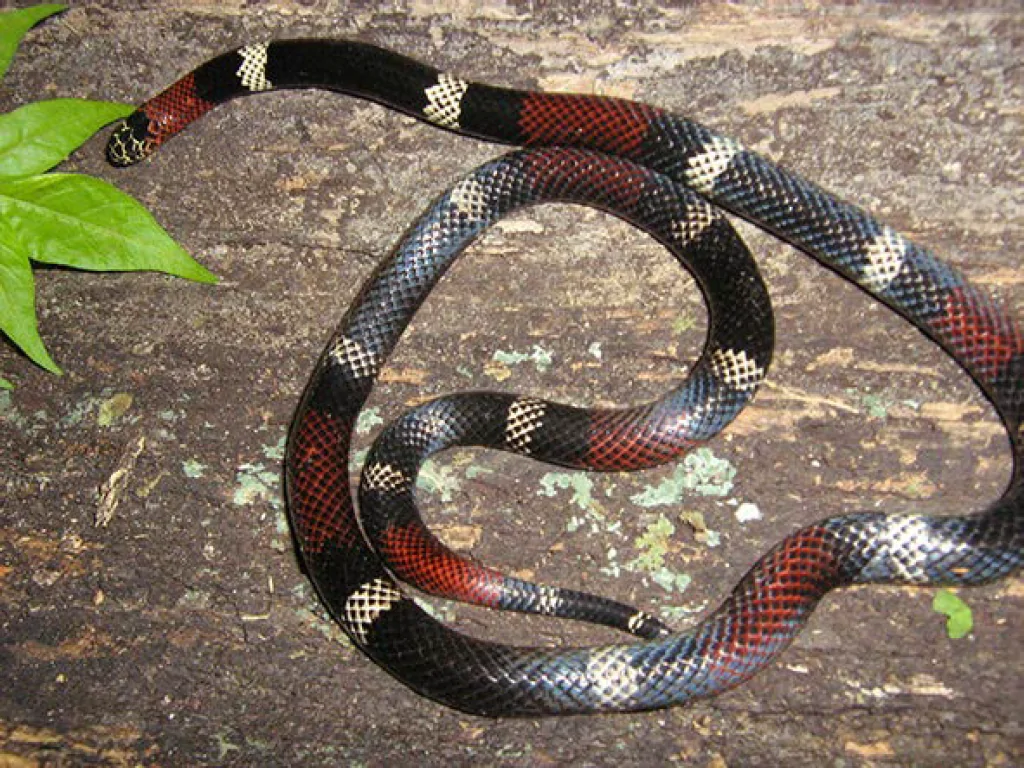
*Micrurus pyrrhocryptus*, province of Córdoba, Argentina (photo credit: Adolfo Rafael de Roodt)

**Figure 2:**
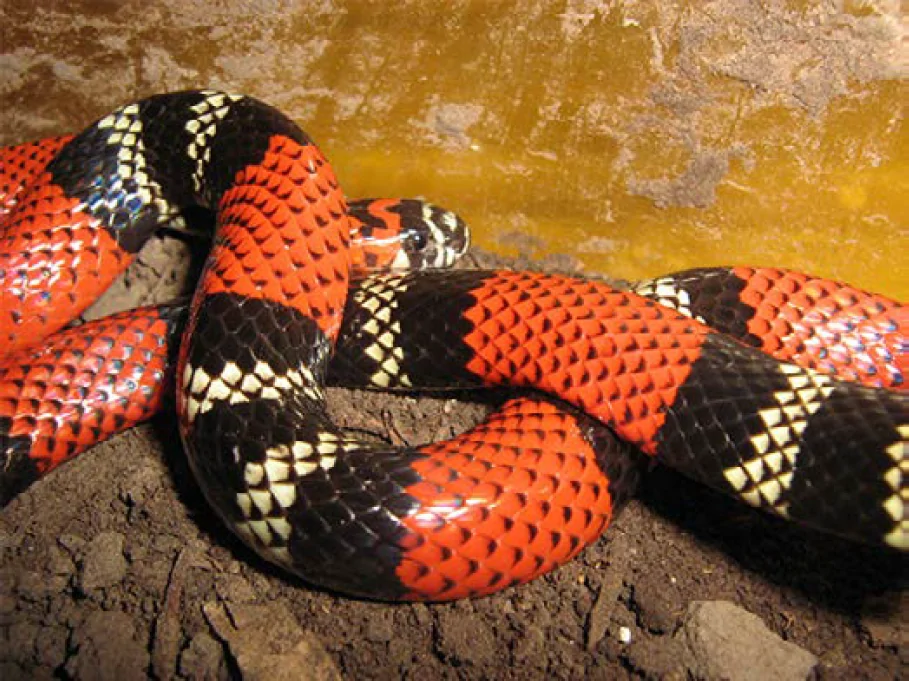
*Micrurus altirostris*, province of Misiones, Argentina (photo credit: Adolfo Rafael de Roodt)

**Figure 3:**
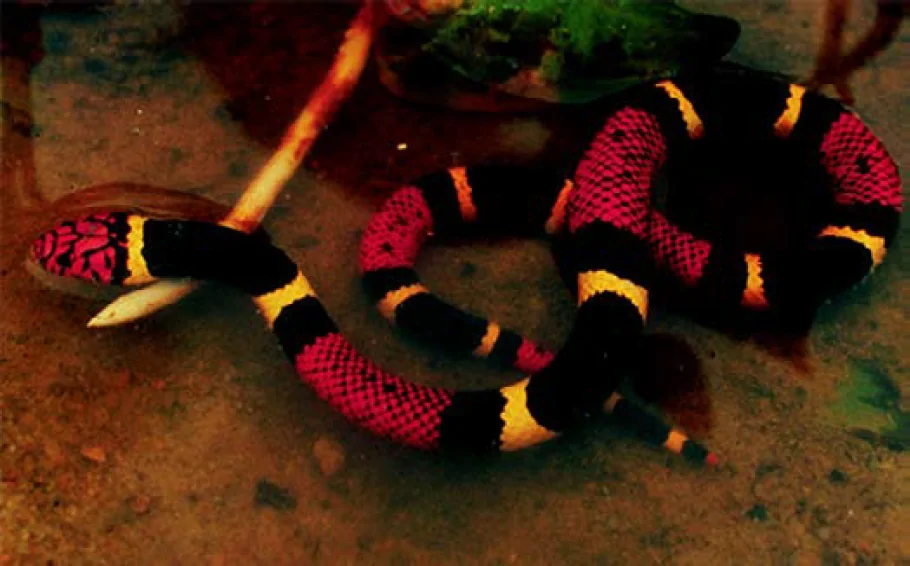
*Micrurus surinamensis*, Letizia, Colombia (photo credit: Juan Silva Haad)

## 2. Materials and methods

### 2.1 Venoms

The venom of *M. pyrrhocryptus* was obtained from specimens collected in various regions of Argentina and kindly provided by Dr. Eduardo Gould (*Fundación de Estudios Biológicos*, Buenos Aires, Argentina). *M. altirostris* venom was donated by Dr. Alejandro Urs Vogt (*Centro Toxicológico de Misiones*, Oberá, Misiones, Argentina). The venom of Colombian *M. surinamensis* was a gift from Dr. Juan Silva Haad (Leticia, Colombia). *M. fulvius* venom from Florida, USA, was sourced from the Miami Serpentarium (Florida, USA) and *M. nigrocinctus* venom from Costa Rica was acquired from the *La Nauyaca Serpentarium* (Cuernavaca, Mexico). All venoms were obtained by manual extraction, immediately vacuum dried or lyophilized, and stored at -20ºC until use.

### 2.2 Animals

All experiments were conducted using 18–22 g CF-1 mice obtained from the Animal Facility of the Instituto Nacional de Producción de Biológicos (INPB). The animals were housed under controlled environmental conditions and received rodent chow and filtered water *ad libitum*. Ethical approval for animal handling was granted by the INPB – ANLIS “Dr. Carlos G. Malbrán”, in agreement with the guidelines of the National Research Council [49,50] and the Federation of European Laboratory Animal Science Associations [30].

### 2.3 Neostigmine (N) and atropine (A)

Neostigmine (Neostigmina Drawer, Laboratory Drawer) was used in 1 mL ampoules at a concentration of 0.5 mg/mL. Atropine (Klonatropina, Klonal Laboratorios), was used in 1 mL ampoules at a concentration of 1 mg/mL. All drugs were used prior to their expiration date.

### 2.4 Antivenom

The AV used was *Antiveneno Elapídico*, produced by the INPB through the immunization of horses with venoms from Argentine *Micrurus* species, primarily *M. pyrrhocryptus* [26], batch No. 133, Exp. Date August 31, 2017. The AV is constituted of F(ab´)_2_ fragments. Although the efficacy of AVs after their expiration date has been documented [17,52,53,59,63], this AV was tested for its physicochemical properties and neutralizing capacity prior to use, confirming that it retained all characteristics declared by the manufacturer.

### 2.5 Determination of the median lethal doses, minimal mortal doses and time to death

The lethal potency of the venoms was determined via the subcutaneous (s.c.) route. Groups of mice (n = 5 per dose level) were injected with various amounts of venom diluted in 0.15 M NaCl, in a final volume of 0.3 mL. Mortality was recorded 24 h post-injection. Lethal potency was defined as 1) the median lethal dose (LD_50_) representing the dose of venom required to kill 50% of the challenged mice within a period of 24 h, and 2) the minimal mortal dose (MMD), the lowest dose of venom that caused 100% mortality in the same period (equivalent to LD100). The venom doses versus mortality plots were analyzed using a sigmoidal dose-response model with a variable slope [14].

Once the MMD was established, groups of mice were injected with 1.2 MMD of each of the venoms, and time to death (from injection to death) was recorded. Group comparisons were performed using the Mann-Whitney test or Student’s *t* test, as appropriate.

### 2.3 Dosages of Neostigmine and Atropine

Several doses of N and A were evaluated based on dose ranges previously used in murine models of intoxication and envenomation involving AChE inhibition [18,34,38,41]. The final selected doses were 0.25 μg/g of N and 2.0 μg/g of A. Both drugs were administered via the intraperitoneal (i.p.) route in a final volume of 0.5 ml/mouse, diluted in 0.15 M NaCl. In combination with AV, the same final volume and diluent were used, with appropriate adjustments for AV volume. The doses of A and N were tested alone and in combination, up to threefold higher than the final experimental dose, in groups of five mice to ensure the absence of toxicity. No adverse effects were observed at these levels. Severe signs of intoxication and mortality (2 out of 5 animals challenged) were only observed at fivefold the selected doses.

### 2.7 Experiments on toxicity reduction

**2.7.1 Effect of different doses of AV with or without N+A**

**2.7.1.1 Experimental groups**

Groups of five mice were injected subcutaneously with 1.2 MMD of each venom, diluted in 0.15 M NaCl to a final volume of 0.3 mL. Fifteen min after envenomation, mice received an intraper-itoneal (i.p.) rescue treatment consisting in AV alone (50 or 100 μL) or AV combined with N+A, diluted in 0.15 M NaCl to a final volume of 0.5 mL. Treatment groups were as follows: group 1) N (5 μg) + A (40 μg); group 2) different doses of AV (50 or 100 μL) plus N (5 μg) +A (40 μg); group 3) AV alone (50 or 100 μL); group 4) venom alone diluted in 0.15 M NaCl (positive control).

**2.7.1.2 Data Interpretation**

The protection provided by each treatment was expressed as the percentage of mice surviving 24 h post-injection. Survival data (the number of deaths and survivors per group) were analyzed using Fisher’s exact test to compare the effectiveness of AV alone versus AV combined with N+A.

**2.7.1.3 Relative delay in death with N+A alone**

Due to the rather different times to death observed following administration of 1.2 MMD of the various venoms, the time to death was recorded for each animal. To assess the potential benefit of treatment with N+A in delaying or preventing death for each venom tested, the relative delay in death (RDD) was defined as the ratio between the mean time to death in mice treated with N+A and the mean time to death in controls (venom only), according to the formula: RDD = (time to death using N+A) / (time to death in controls).

**2.7.1.4 Analysis of survival data**

Median survival outcomes from the various experiments were analyzed using linear and non-linear regression models. The median values derived from sigmoidal dose-response analyses were considered Theoretical Median Doses (T-MED). Additionally, the ratios between the T-MED values obtained with and without N+A treatment, were calculated and analyzed.

**2.7.2 Effect of N+A on the rescue Median Effective Dose of the AV on** *M. pyrrhocryptus* **and** ***M. altirostris*** **venoms**

In order to evaluate whether the use of N+A alters the Median Effective Dose (ED_50_) of the AV against envenomation by *M. pyrrhocryptus* and *M. altirostris* venoms, rescue ED_50_ (r-ED_50_) values were estimated in ED_50_ assays with and without N+A co-administration. Groups of 5 mice were s.c. injected in the dorsal region with 1.2 MMD of the venoms in a final volume of 0.3 mL of 0.15 M NaCl. After fifteen minutes, the mice received an i.p. injection of different doses of AV alone or AV plus N+A, in a final volume of 0.5 ml/mouse, in 0.15 M NaCl. Mice were monitored and after 24 h survival deaths were recorded. The rescue median effective dose (r-ED_50_) was defined as the AV dose that protected 50% of the mice [54] and was estimated using a sigmoidal dose-response model with variable slope [14].

### 2.8 Statistics

The distribution of the data was assessed using the Kolmogorov-Smirnov test. For comparison of groups, Mann-Whitney or Student’s *t* test were used. For multiple comparison analysis, ANOVA or Kruskal-Wallis tests with Bonferron's or Dun's adjustment tests were used. Differences in frequencies were analyzed using Fisher’s exact test. The dose-response experiments were analyzed by linear regression or non-linear regression using a sigmoidal dose-response model with variable slope. All statistical analyses were performed using the software Prism 6.0 (GraphPad Inc., CA, USA) for Mac. A p-value ≤ 0.050 was considered statistically significant.

## 3 Results

### 3.1 Lethal potencies

The s.c. LD_50_s, LD_100_s (MMDs) of the venoms used in this study were 31 μg and 45 μg for *M. pyrrhocryptus*, 26 μg and 30 μg for *M. altirostris*, 28 μg and 40 μg for *M. surinamensis*, 27 μg and 57 μg for *M. nigrocinctus* and, 22 μg and 40 μg for *M. fulvius* venoms. These doses and the venom masses that represent the 1.2 MMD challenge doses used in this study, are detailed in Table I.

**Table I:** Median lethal dose (LD_50_), minimal mortal dose (MMD or LD_100_) and challenge dose (1.2 MMD) of the

Venin	Doses toxiques		
	DL_50_ s.c. (μg/souris)	DL_100_ s.c. (DMM) (μg/souris)	Dose de provocation (1,2 DMM) (μg/souris)
*M. pyrrhocryptus*	31 (28 to 35)*	45	54
*M. altirostris*	26 (25-27)*	30	36
*M. surinamensis*	28 (25 to 30)*	40	48
*M. nigrocinctus*	27 (21 to 35)*	57	68
*M. fulvius*	22 (19 to 27)*	40	48

les valeurs entre parenthèses correspondent aux intervalles de confiance à 95 %

### 3.2 Time to death of mice injected with venom with or without treatment with N+A

The venom of *M. surinamensis* caused the most rapid lethality, with a mean time to death of 25 ± 6 min while *M. fulvius* venom resulted in the slowest progression to death, with a mean time of 157 ± 35 min (ANOVA-Bonferroni p < 0.025 and p < 0.0001 for *M. surinamensis* and *M. fulvius* respectively). The mean time to death for the remaining venoms were 43 ± 6 min, 47 ± 11 min and 54 ± 9 min for *M. nigrocinctus*, *M. pyrrhocryptus* and *M. altirostris* venoms respectively (Table II). The RDD is shown in Figure 4.

**Figure 4:**
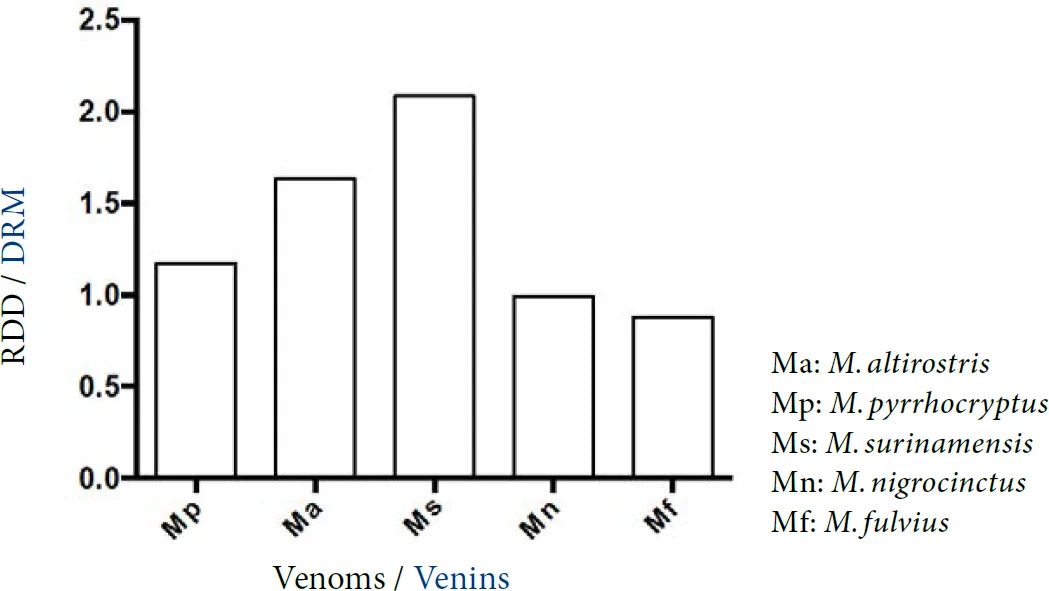
Relative delay in time to death (RDD) following treatment with N+A. Bars represent the ratio between the time to death of mice injected with V and treated with N+A, and the time to death of the controls injected with V alone (RDD = V + N+A/V alone)

**Table II:** Times of death caused by the injection of 1.2 MMD of the different venom and with and without the injection of N+A

Venin	Traitement	Time of death (min)				n	p
		Mean ± SD	Mediane	Minimum	Maximum		Statistic
*M. pyrrhocryptus*	Venin seul	47 ± 11	48	30	75	22	< 0.05 (U = 174,5; t = 2.09)*
	Venin plus N+A	55 ± 15	50	45	114	24	
*M. altirostris*	Venin seul	54 ± 9	54	41	70	17	< 0.001 (U = 20.5)
	Venin plus N+A	76 ± 14	78	51	101	15	
*M. surinamensis*	Venin seul	25 ± 6	24	18	42	18	< 0.001 (U = 0.0)
	Venin plus N+A	60 ± 27	53	43	122	8	
*M. nigrocinctus*	Venin seul	43 ± 6	42	34	56	13	0.69 (U = 76.5)
	Venin plus N+A	43 ± 4	42	36	51	13	
*M. fulvius*	Venin seul	157 ± 35	151	110	217	12	0.29 (U = 53.5)
	Venin plus N+A	132 ± 46	147	59	190	12	

n : nombre d'animaux; U : valeur (test de Mann-Whitney); * : test t de Student); dans ce cas (venin de *M. pyrrhocryptus*), les différences entre les groupes ont été analysées à l'aide des méthodes du test t de Student et du test de Mann-Whitney

The combination of N+A did not protect mice of the challenge dose, but delayed the time of death of *M. surinamensis, M. altirostris* and *M. pyrrhocryptus* venoms. Only in the case of *M. altirostris* the survival of 2 mice out 15 treated with venom and N+A was observed (Table II).

### 3.3 Survival of mice injected with venom and rescued with AV alone or with AV plus N+A

The combination of the treatment with AV plus N+A resulted in improved outcomes in enven-omations by South American *Micrurus* species.

**3.3.1** ***M. pyrrhocryptus*** **venom**

The addition of N+A to AV resulted in better outcomes than treatment with AV alone (Table III; Appendix 2). Among mice challenged with this venom, survival was higher in the group treated with 100 μL of AV plus N+A (21/25 mice survived with the addition of N+A, compared to 11/25 in the AV-alone group) or using 50 μL of AV (13/25 survivors with N+A vs 7/25 using AV alone). Fisher’s exact test confirmed a statistically significant improvement with 100 μL of AV plus N+A compared to 100 μL of AV alone (p = 0.0072) (Fig. 5; Fig. 6; Appendix 2). Although protection appeared higher with 50 μL AV plus N+A compared to 50 μL of AV alone, in this assay the difference did not reach statistical significance (p = 0.0833). Nevertheless, statistical differences were observed when the protection provided by this dose of AV plus N+A regarding AV alone in ED_50_ experiments (n: 30 mice) were analyzed (p = 0.0352) (Appendix 3.a). This difference when all data using a dose of 50 μL of AV alone or plus N+A (fixed doses + ED_50_ assays, n: 80 mice) were analyzed (p = 0.0032) (Appendix 3.b).

**Figure 5:**
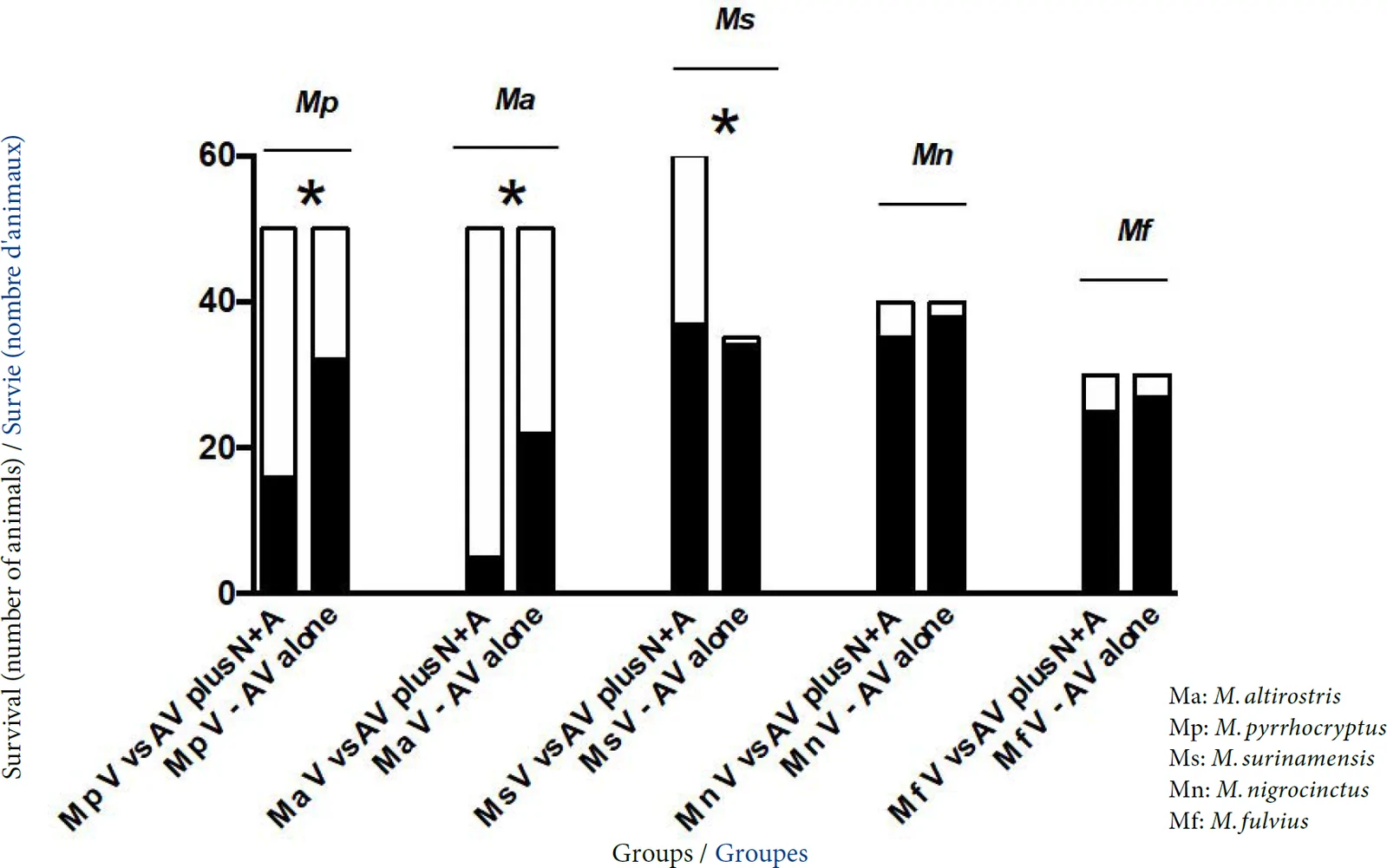
General response to the different treatments. Survival and mortality outcomes of mice challenged with 1.2 MMD of venom and treated with either AV alone or AV plus N and A (doses up to 250 μL were included). The bars represent the cumulative results from all experiments. Black portions indicate deceased mice, and white portions indicate surviving mice. Asterisks indicate statistically significant differences (p < 0.05, Fisher’s exact test) comparing survival and mortality rates between mice treated with AV alone and those receiving AV plus N+A

**Figure 6:**
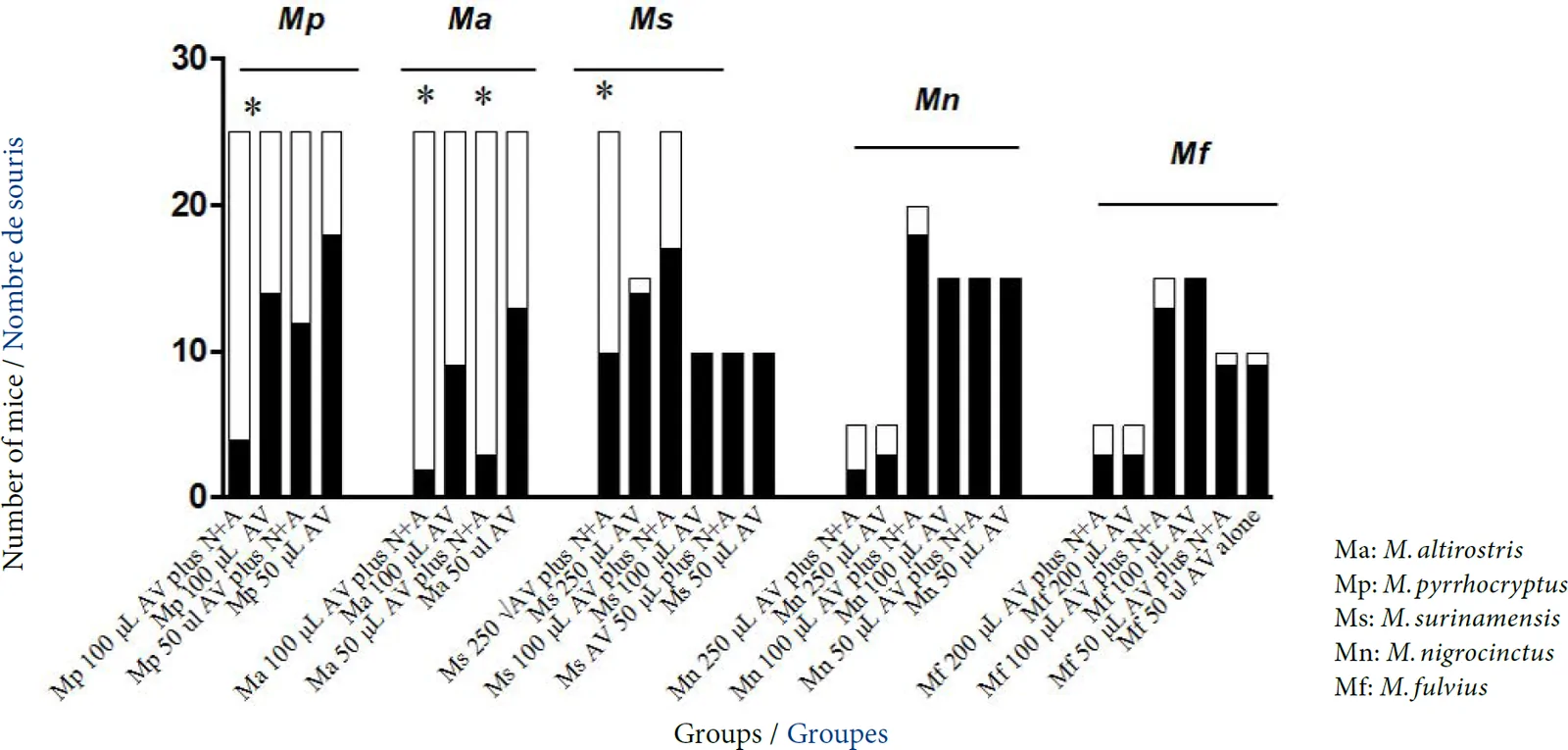
Response to different doses of AV with or without N+A. Survival and mortality outcomes in mice challenged with 1.2 MMD of venom (V) and treated with different doses of either AV alone or with AV plus N+A were compared using Fisher’s exact test. Bars represent cumulative data per dose. Black portions indicate deceased mice, and white portions indicate surviving mice. Asterisks indicate statistically significant differences (p < 0.05).

**Table 3:** Percentual survival of mice challenged with 1.2 MMD of venom and rescued with different fixed doses of AV alone or combined with N+A

Venin	Traitement	Dose AV (μL)	Protection (%)			Nombre d'expériences
			Médiane	Minimum	Maximum	
*M. pyrrhocryptus*	AV alone	50	20	0	60	5
		100	40	20	60	5
		250	100	*	*	1
	AV plus N+A	50	60	0	100	5
		100	80	60	100	5
		250	100	*	*	1
*M. altirostris*	AV alone	50	60	0	60	5
		100	60	40	80	5
		250	100	*	*	1
	AV plus N+A	50	100	60	100	5
		100	100	80	100	5
		250	100	*	*	1
*M. surinamensis*	AV alone	50	0	0	0	2
		100	0	0	0	2
		250	20	0	20	3
	AV plus N+A	50	0	0	0	2
		100	20	0	80	5
		250	60	0	100	5
*M. nigrocinctus*	AV alone	50	0	0	0	3
		100	0	0	0	4
		250	40	*	*	1
	AV plus N+A	50	0	0	0	3
		100	10	0	20	4
		250	40	*	*	1
*M. fulvius*	AV alone	50	10	0	20	2
		100	0	0	0	3
		200	40	*	*	1
		400	60	*	*	1
	AV plus N+A	50	10	0	20	2
		100	20	0	20	3
		200	40	*	*	1
		400	100	*	*	1

There were used 5 mice by experiment

**3.3.2** ***M. altirostris*** **venom**

The adding of N+A to the treatment with AV improved the survival. Among mice challenged, the use of the combination showed a better outcome (Table III; Appendix 2), when using 100 μL of AV, 16/25 mice survived in the AV-alone group while 23/25 survived in the AV plus N+A group. When the dose of AV was 50μL, the combination of AV plus N+A protected 22/25 while the use of AV alone only protected 12/25 challenge mice. Fisher’s exact test showed that combining either 100 or 50 μL of AV with N+A offered significantly better protection than AV alone (p = 0.0374 and 0.0054, respectively) (Fig. 5; Fig. 6; Appendix 2).

**3.3.3** ***M. surinamensis*** **venom**

Overall, AV alone protected just 1 of 15 mice at a dose of 250 μL (1/15), while 100 or 250 μL of AV plus N+A protected 8/25 and 15/25 respectively. No protection was observed with 50 μL of AV plus N+A (0/10) (Table III). Fisher’s exact test showed that the best outcome was achieved using 250 μL of AV plus N+A (p = 0.0009). The use of 100 μL of AV and N+A, although seemingly better, did not differ significantly from AV alone (p = 0.0734) (Fig. 5; Fig. 6; Appendix 2).

**3.3.4** ***M. nigrocinctus*** **venom**

No meaningful differences in survival were observed between AV alone and AV combined with N+A; 2/40 versus 5/40 mice survived (n=80) (Table III; Fig. 6). When each dose was analyzed separately using Fisher’s exact test, no statistically significant differences were found (p > 0.4 in all cases) (Fig. 5; Fig. 6; Appendix 2).

**3.3.5** ***M. fulvius*** **venom**

No statistically significant differences in survival were observed between treatment with AV alone or AV combined with N+A. When considering the different doses, some degree of protection was observed (Table III), but Fisher’s exact test showed no significant differences (p > 0.4 in all cases) (Fig. 5; Fig. 6; Appendix 2).

### 3.4 Variation in the Rescue Median Effective dose of AV with or without N+A

The addition of N+A to the AV treatment en-hanced its neutralizing potency against both venoms, although the magnitude of the improvement varied. For *M. altirostris* venom, the ED_50_ of AV alone was 55 μL (95% CI: 46–67 μL, R^2^ = 0.9781), which decreased to 16 μL (9–28 μL, R^2^ = 0.7655) when combined with N+A, reflecting a 3.4-fold increase in neutralizing potency. In contrast, for *M. pyrrhocryptus* venom, the ED_50_ of AV alone was 94 μL (81–108 μL, R^2^ = 0.7727), and this was reduced to 59 μL (47–74 μL, R^2 =^ 0.8273) with the addition of N+A, corresponding to a 1.6-fold improvement (Fig. 7).

**Figure 7:**
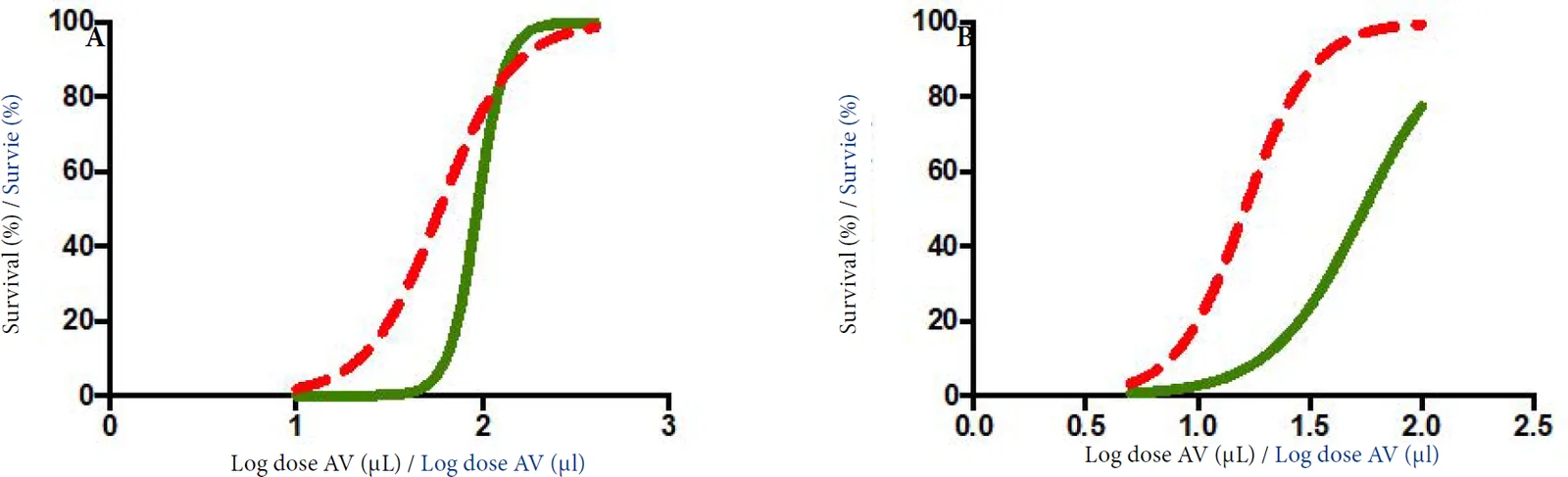
Median effective dose of AV, administered alone or in combination with N+A, in rescue experiments. AV or AV plus N+A was injected intraperitoneally 15 min after subcutaneous injection of 1.2 minimal mortal doses (MMD) of the respective venoms. The solid line represents the response to AV alone, while the dashed line represents the response to AV plus N+A. In both cases, the addition of N+A reduced the dose of AV required to rescue 50% of challenged mice. A. Against *M. pyrrhocryptus* venom, the ED_50_ of AV alone was 94 μL (95% CI: 81 – 108 μL, R^2^ = 0.7727), while the addition of N+A reduced the ED_50_ to 59 μL (47–74 μL, R^2^ = 0.8273), increasing neutralizing potency by 1.6-fold. B. For *M. altirostris* venom, the ED_50_ of AV alone was 55 μL (46–67 μL, R^2^ = 0.9781), reduced to 16 μL (9–28 μL, R^2^ = 0.7655) when combined with N+A, corresponding to a 3.4-fold increase in neutralizing potency.

### 3.5 Analysis of the doses used and protection

In all cases, treatment improved the outcome of experimental envenomations to varying degrees. However, statistically significant differences were observed only for *M. altirostris* and *M. pyrrhocryptus* venoms and for *M. surinamensis* venom but only using high doses of AV (Fig. 5 and 6). A preliminary analysis using linear and non-linear regression of median protection values against AV doses confirmed this trend (Appendix 4 and 5). The data indicate that the addition of N+A allows for a reduction in the required AV dose to achieve the same level of protection across all venoms tested (Table III; Fig. 5; Fig. 8; Appendix 4 and 5). For the neutralization of *M. pyrrhocryptus* and *M. altirostris* venoms in particular, although the ED_50_ and T-MED values differed by nearly two-fold, their 95% confidence intervals overlapped (Fig. 7; Appendix 6).

**Figure 8:**
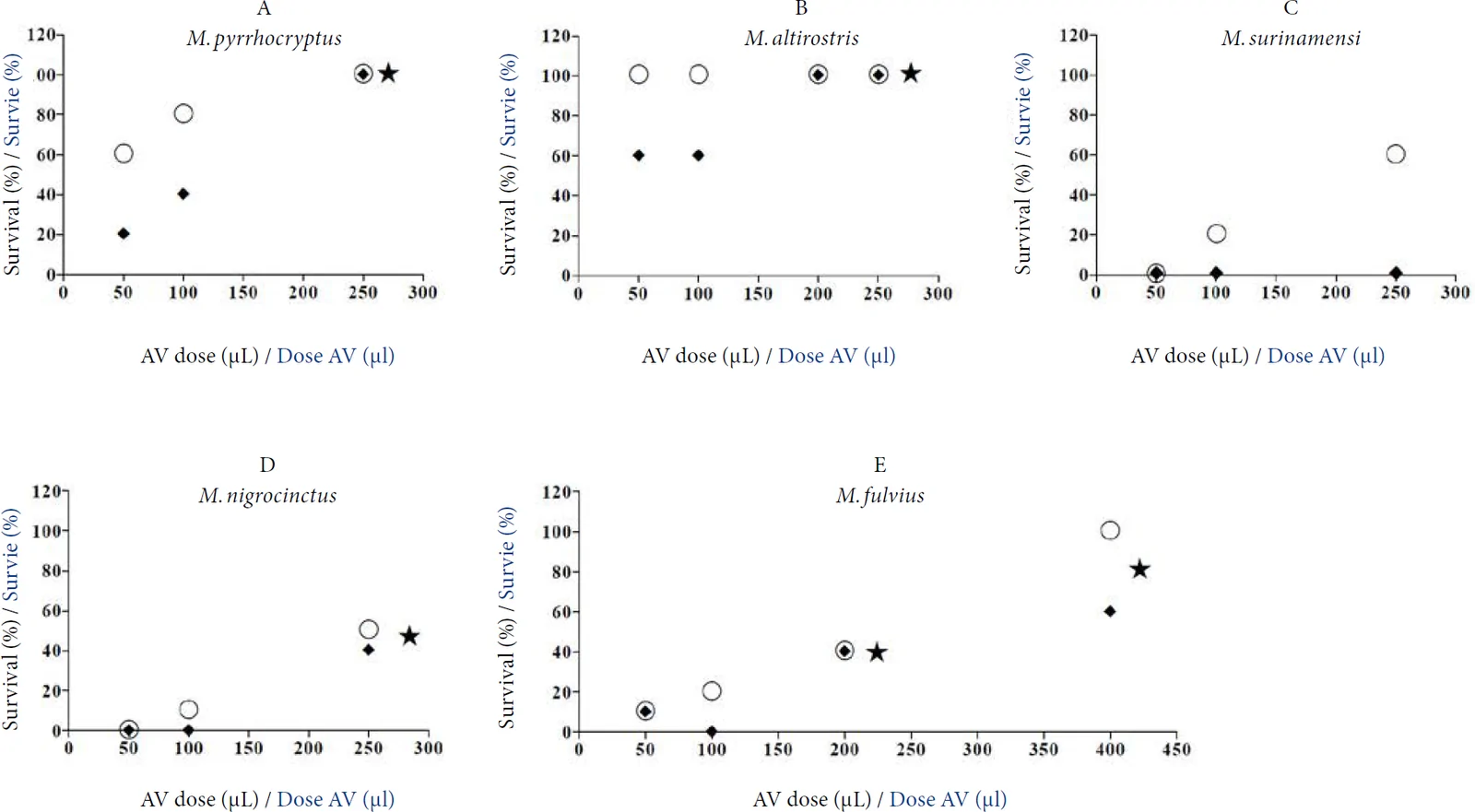
The median survival values from the different rescue experiments are plotted as percentages against the corresponding AV doses. Treatments using AV alone are represented by black rhombi: ♦ and treatments using AV plus N+A by white circles: O*.* Stars (*) denote data points for which only a single experiment was performed (highest doses).

## 4. Discussion

Envenomation by coral snakes is uncommon, but when it does occur it constitutes a medical emergency for which the only effective treatment is the administration of specific AV [45,46]. This AV is difficult to produce and is of limited availability, even in countries with local manufacturing capabilities [10,11,23,25,27].

The AChEIs alone or combined with AVs have been used for the treatment of envenomation by other elapids [4,37,48]. Despite that some experimental benefits were observed on envenomation caused by venoms with both pre and postsynaptic neurotoxins [76], in envenomation by other elapids, clinical experience shows absence or low benefits. In envenomations caused by venoms with presynaptic neurotoxins like *Bungarus* or *Oxyuranus* species [2,62,65,72] the benefit is lower or absent regarding their use on envenomations with venoms with predominantly postsynaptic neurotoxins like *Naja* [29,36,56,73,74,76]. In the case of coral snakes, the use of AChEIs is particularly recommended in case of envenomation by snakes whose venom consists exclusively of postsynaptic neurotoxins [9,10,46,67].

As previously mentioned, the use of AChEIs has been proposed as a therapeutic approach for *Micrurus* envenomations [32,67,69,70,71], nevertheless, some *Micrurus* venoms, such as those of *M. corallinus* and *M. fulvius,* also cause presynaptic neurotoxicity via β-neurotoxins [19,66,67]. The action of these β-neurotoxins, along with certain non-neurotoxic PLA_2_s, involves additional mechanisms that can cause lesions in cellular membranes, potentially complicating the course of envenomation [4,6,47]. Due to this significant variability, AChEIs are not always effective and their role may be more appropriate as an adjunctive rather than a primary treatment modality [8,10,11].

As mentioned above, the outcome of mice treated is related with the composition of the venoms, especially by the type of neurotoxins. The addition of N+A might have been expected to yield good results in venoms with higher proportions of α-neurotoxins, such as *M. surinamensis* (95.4%) [60] and *M. altirostris* (79.5%) [19]. In the case of *M. altirostris* the benefits were clear but in the case of *M. surinamensis* the results may be possibly related to the differential characteristics of this venom [55,64], the non specificity of the antiven-om as observed in previous studies [26,27] and the difficult in neutralize this venom inclusive with specific antivenom [26]. Nonetheless, the use of N+A at high doses improves the effectiveness of the AV against this venom.

However, despite the presence of 3-FTxs in *M. nigrocinctus* venom (38%) [31] and in *M. fulvius* venom (24.9%) [44,66], the improvement was lower than that observed on *M. pyrrhocryptus* venom, with 27%. The limited or absent effectiveness of the combination AV plus N+A against envenomations by *M. fulvius* and *M. nigrocinctus* at the doses tested may be due to the presence of myotoxic and neurotoxic PLA_2_s in *M. fulvius* [44,66], over 58% of the venom mass, and myotoxic PLA_2_s representing 48% of the venom mass in *M. nigrocinctus* venom [25,35,44,57,66] (Appendix 7).

In experimental envenomations using *M. fulvius* venom, good neutralization has been observed using PLA_2_ inhibitors [39,40], which reinforces the important role of β-neurotoxicity in this envenomation. On the other hand, despite the high concentration of PLA_2_ in the venom of *M. nigrocinctus*, its β-neurotoxicity does not appear to play a significant role in envenomation [31], unlike its myotoxicity [25,31,57]. The venom of *M. pyrrhocryptus* has low experimental myotoxicity [12,25], activity not observed in *M. surinamensis* and *M. altirostris* venoms [25]. Since N+A does not counteract these toxic mechanisms, this may explain the results obtained in these envenomations in the present study.

In Brazil, the use of AChEIs is suggested as an ancillary intervention when AV obtaining is un-available, delayed, or insufficient, or in patients who do not improve after receiving the highest recommended doses of AV, or who experience delayed recovery or even recurrent systemic neurotoxic manifestations [9,10]. *In vitro* studies have shown that the neuromuscular blockade induced by various *Micrurus* species, e.g., *M. altirostris, M. laticollaris, M. nigrocinctus, M. pyrrhocryptus* and *M. spixii*, involves interactions with post-synaptic nicotinic receptors. However, reversal by N is typically transitory and often minimal or discrete (generally <30%), offering no sub-stantial long-term protection against progressive neuromuscular blockade. Complete reversal of postsynaptic neurotoxicity has only been achieved with the administration of AV [9,10,11]. Magalhães Camargo *et al.* demonstrated that treatment with N transiently reversed neuromuscular blockade following 50% inhibition induced by *M. pyrrhocryptus* venom, but complete and irreversible blockade occurred after 60 min. This may explain the limited protection afforded by N+A alone in one dose and the improved survival observed when combining N+A with AV [1,4, 33,38].

In the envenomation by snakes it is very important the rapid instauration of the specific treatment, but in the case of neurotoxic envenomations this is specially important since the best effectiveness is obtained when the AV avoid the union of the toxins to their targets [3,16]. The same occurs with the use of N, which would provide better results in the beginning of the envenomation. In Brazil there were observed good outcomes in the envenomation by *Micururus* using the combination of AV plus N+A, when applied 2 hours and 5 hours after the bite but, using two doses of N+A [11]. Nevertheless efforts must be directed to the early use of the N+A.

Unfortunately dose-response studies conducted to compare ED_50_s of the AVs alone or combined with N+A could only be done for *M. pyrrhocryptus* and *M. altirostris* venoms. We did not have enough material to determine ED_50_s for all venoms in dedicated experiments. We hope to obtain sufficient venom and AV in the future to conduct further studies.

## 5 Conclusions

In this study, we observed improved outcomes when combined N+A with AV, being the AV used non species specific for 4 of 5 of the venoms studied. For the three South American coral snake venoms tested, this combination allowed the use of lower AV doses compared to AV alone. In these scenarios, the combination may prove useful when increasing AV dosage is not feasible or when other complementary measures, such as respiratory assistance [11], are unavailable.

We did not observe positive outcomes in the cases of *M. nigrocinctus* and *M. fulvius* envenomations. However, it should be noted that the AV used was neither specific nor closely related to these venoms, and previous studies have shown low protection with this AV against these venoms [26,27]. Further studies on the combination AV plus N+A (or other AChEIs) using specific or more closely related AVs, or higher doses of paraspecific AVs, may yield more conclusive results on the potential benefit of using AChEIs in these envenomations.

The combination of AChEIs with AVs, and potentially with PLA_2_ inhibitors, should be explored further as a strategy to improve existing treatments or to provide alternative therapeutic options for these rare but very serious envenomations, particularly in regions where AV may be scarce or unavailable.

Our findings agree with several observations on the usefulness of the combination N+A in some Elapidae envenomations and although experimental, show the potential help of the combination of N+A plus antivenom in this type of envenomations. Although our study, made in mice, not necessarily can be extrapolated for the therapeutic in humans, the results strongly provide important information on the use of N+A plus antivenom in envenomations by species *Micrurus* with venoms of different venom composition.

To our knowledge, this is the first *in vivo* study on the usefulness of the combination of an anti-*Micrurus* AV with AChEIs in the treatment of envenomation by the southernmost American coral snakes, *M. pyrrhocryptus and M. altirostris*.

## Funding

This research received no specific grant from any funding agency in the public, commercial, or not-for-profit sectors.

## Authors’ contributions

ARdR designed the study, DD, FAMG, MAD and ArdR conducted the fieldwork, ARdR conducted the data analysis, CFD and ARdR supervised the study, ArdR and DD reviewed and edited the final version. All authors read and approved the final manuscript.

## Conflicts of interest

The authors declare no conflicts of interest.

## Appendixes

**Appendix I:**
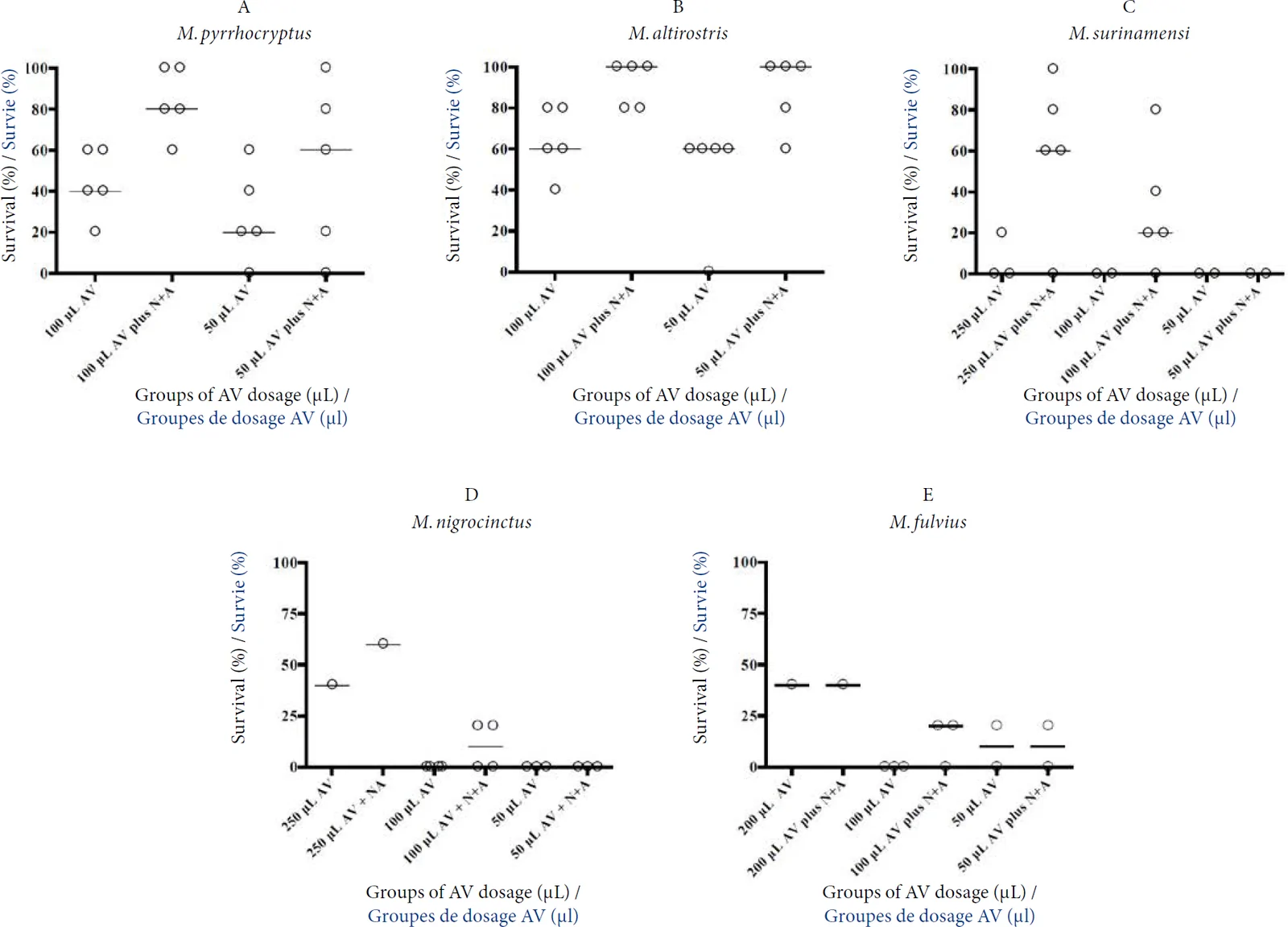
Survival (percentage) of mice challenged with 1.2 MMD of different *Micrurus* venoms and treated with AV alone or a combination of AV plus N+A A: *M. pyrrhocryptus* venom B: *M. altirostris* venom C: *M. surinamensis* venom D: *M. nigrocinctus* venom E: *M. fulvius* venom Bars indicate the median survival for each group of experiments. Each point represents one experiment

**Appendix II:**
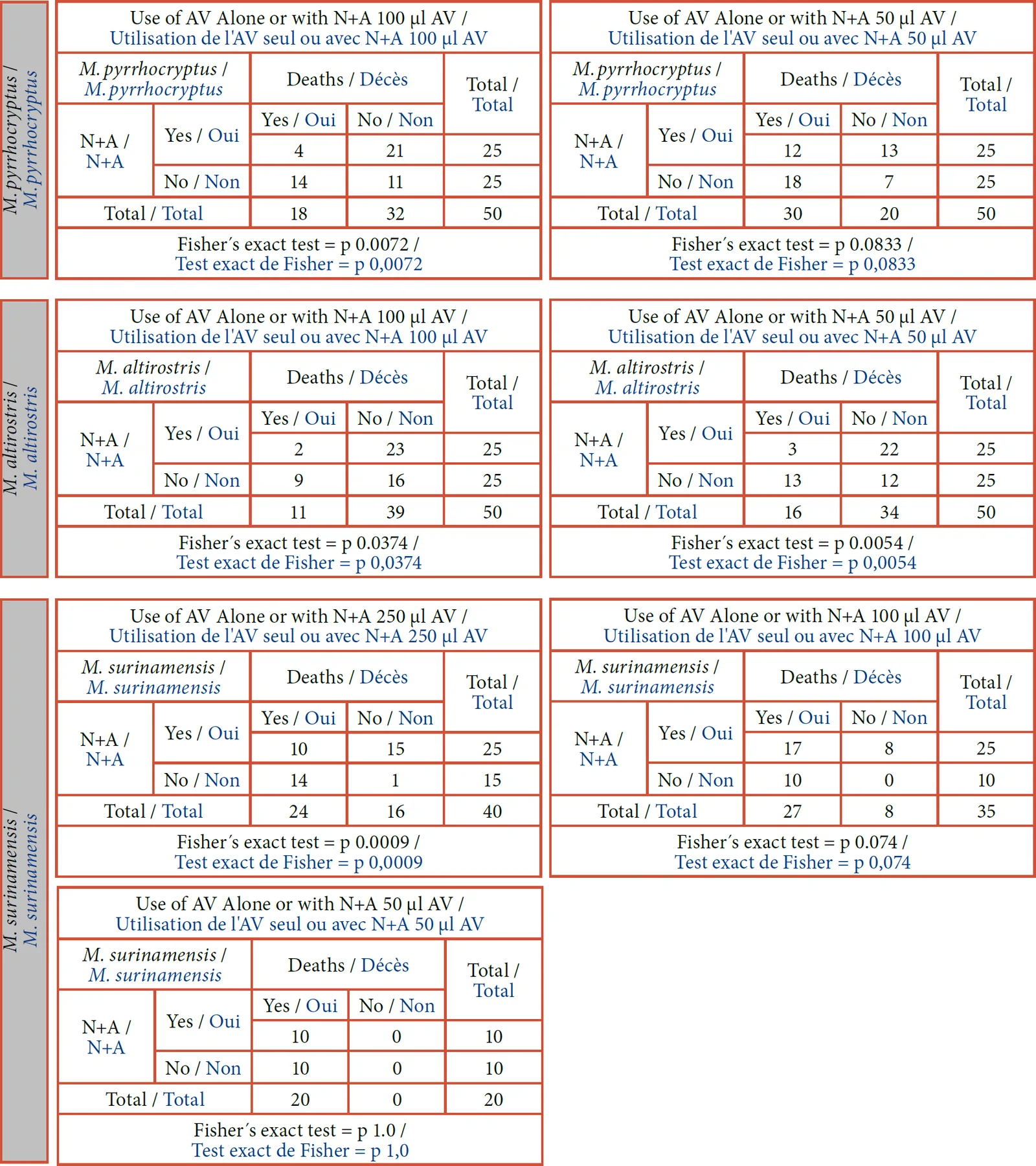
Fisher exact test of the survival of mice challenged with the different venoms and treated with different doses of AV injected alone or in combination with N+A (neostigmine plus atropin).

**Figure:**
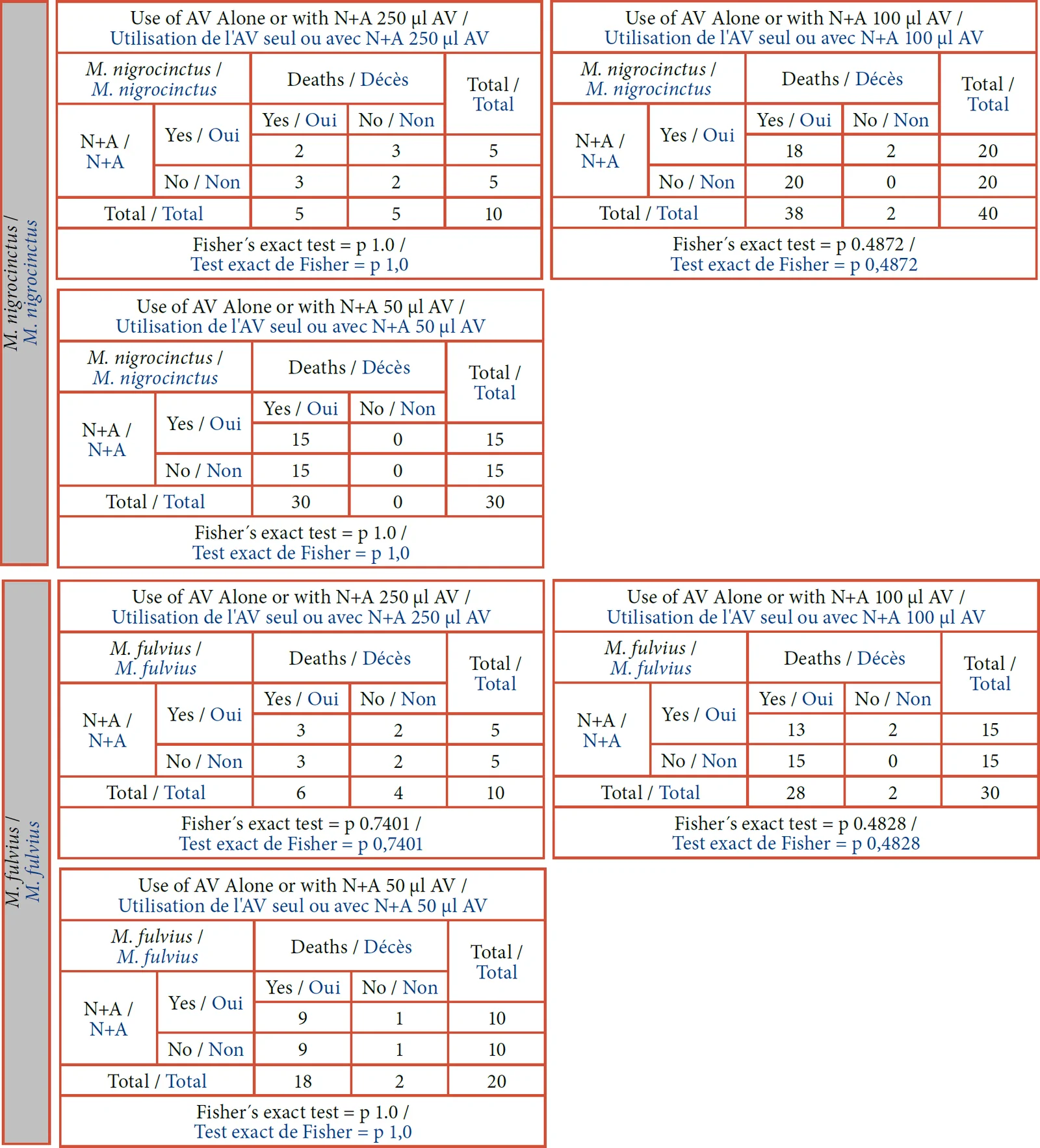

**Appendix III:**
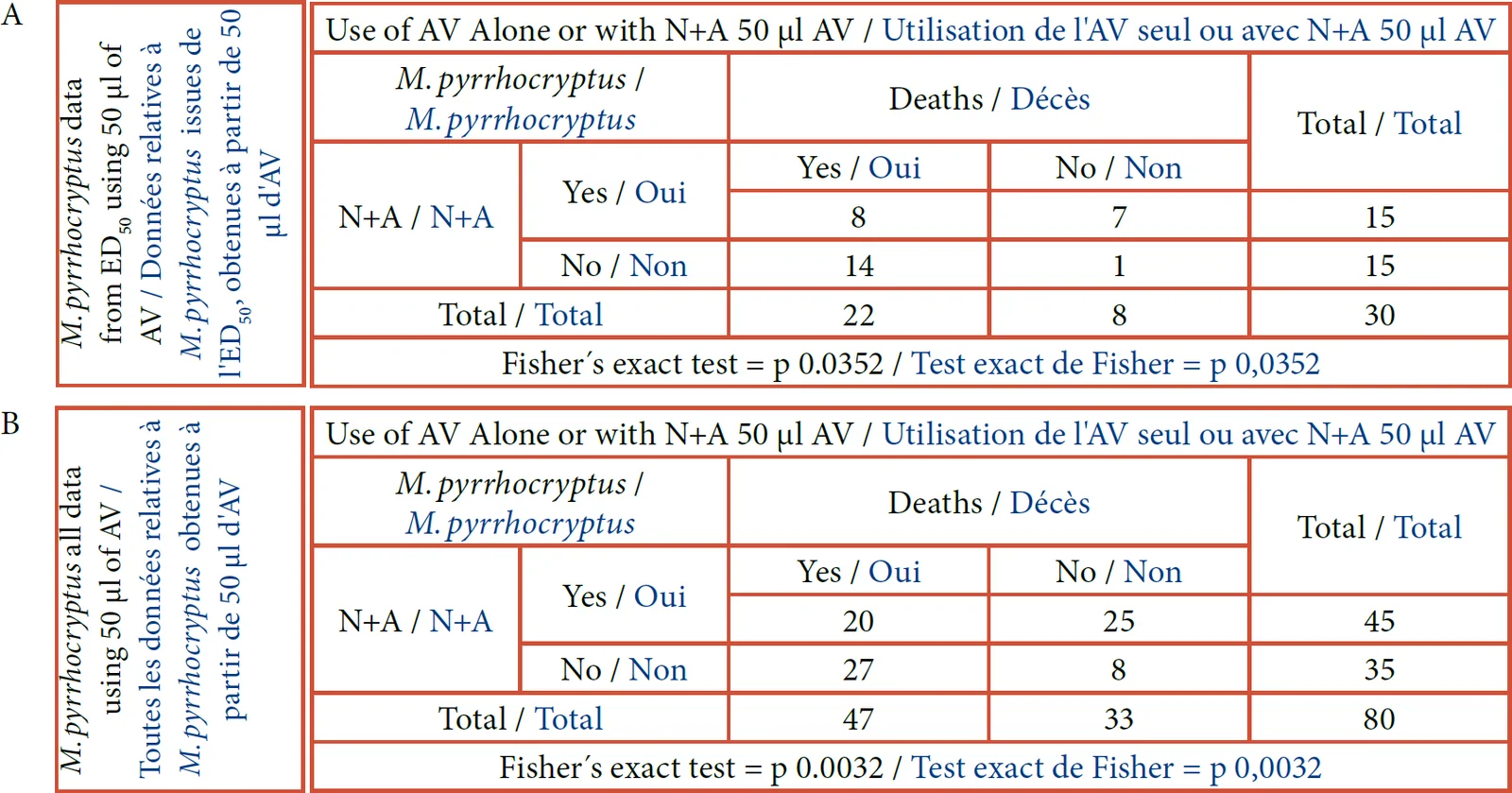
A. Survival of mice challenged with *M. pyrrhocryptus* venom and treated with 50 μL of AV alone or with the addition of N+A. Data from the ED_50_ studies. B. Survival of mice challenged with *M. pyrrhocryptus* venom and treated with 50 μL of AV alone or with the addition of N+A. Data from the ED_50_ (n=30) studies and from the challenge with fixed doses (n= 50)

**Appendix IVa:**
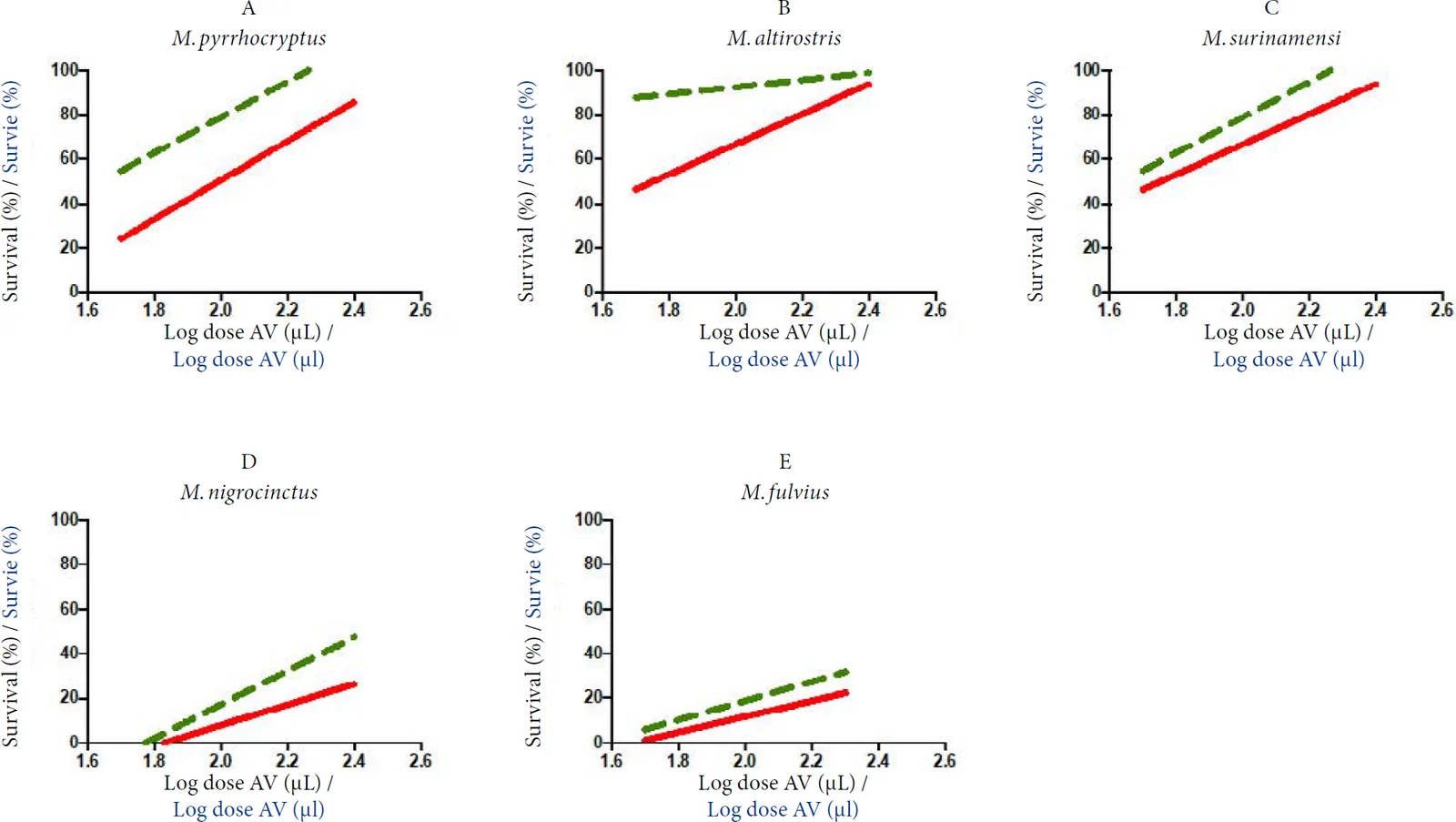
Linear regression of the relationship between log transformed AV doses (in μL) and median survival (%) of mice challenged. Solid lines represent the response to AV alone, while the dashed lines represent the response to AV plus N+A. In all cases, the combination of AV with N+A, required lower AV doses than using the AV alone to produce the same effect.

**Appendix IVb:**
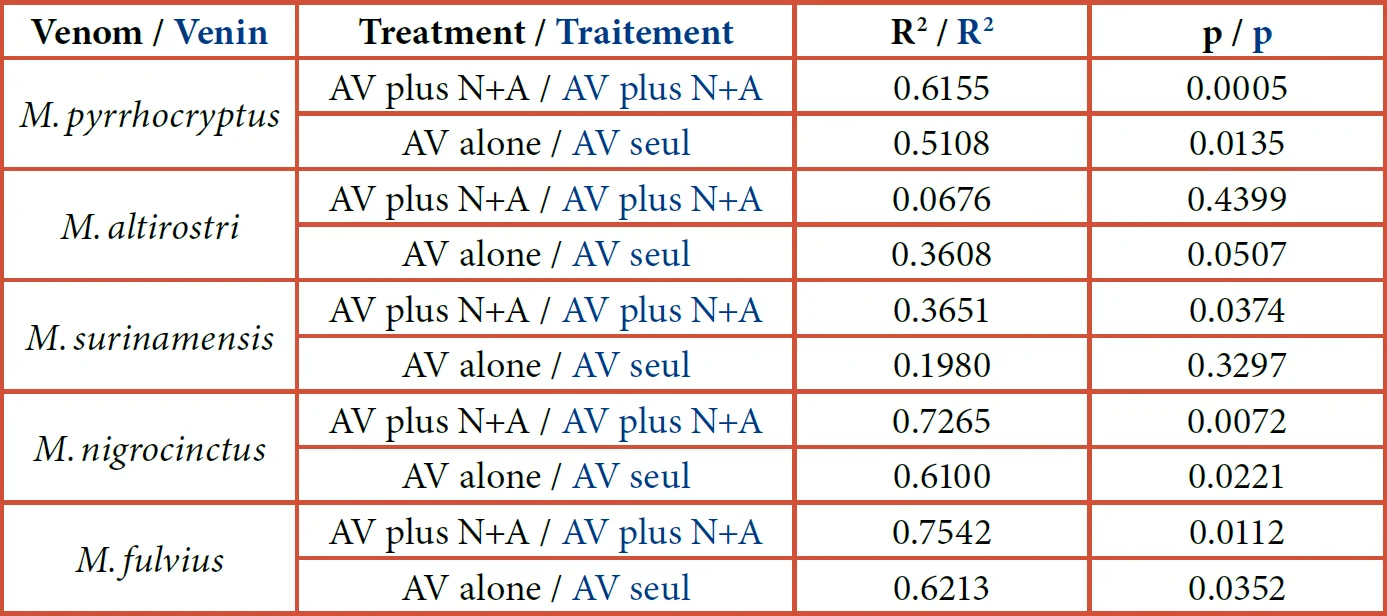
Regression values and p-values for the results presented in 4.a. In the case of *M. altirostris* and *M. surinamensis* venoms, the low R2 and p-values are attributable to the high (for *M. altirostris*) or low (for *M. surinamensis*) response observed at the AV doses used.

**Appendix V:**
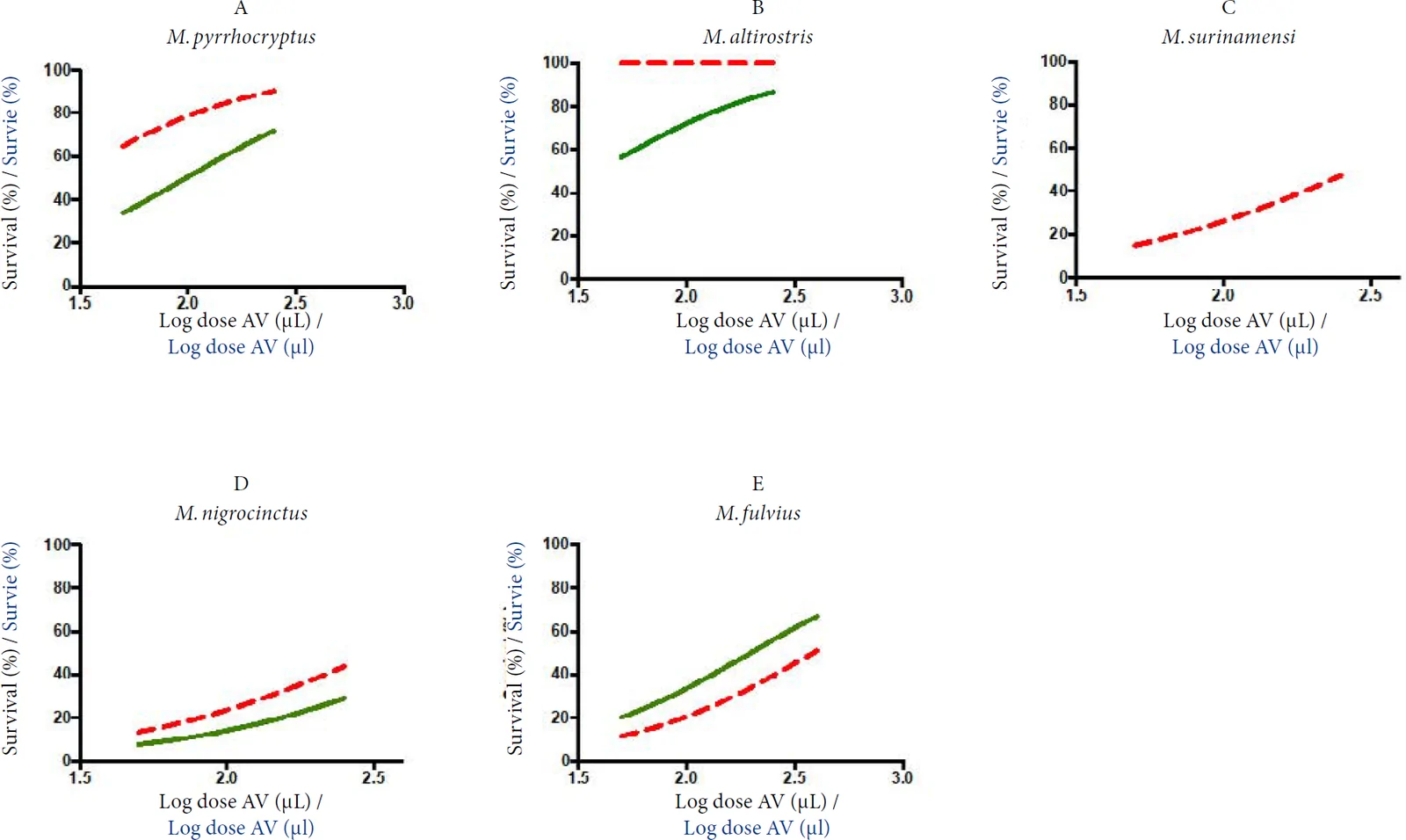
Non-Linear regression of the relationship between log transformed AV doses (in μL) and median survival (%) of mice challenged. Solid lines represent the response to AV alone, while the dashed lines represent the response to AV plus N+A. In all cases, lower AV doses were required to produce the same effect (Table III).

**Appendix VI:**
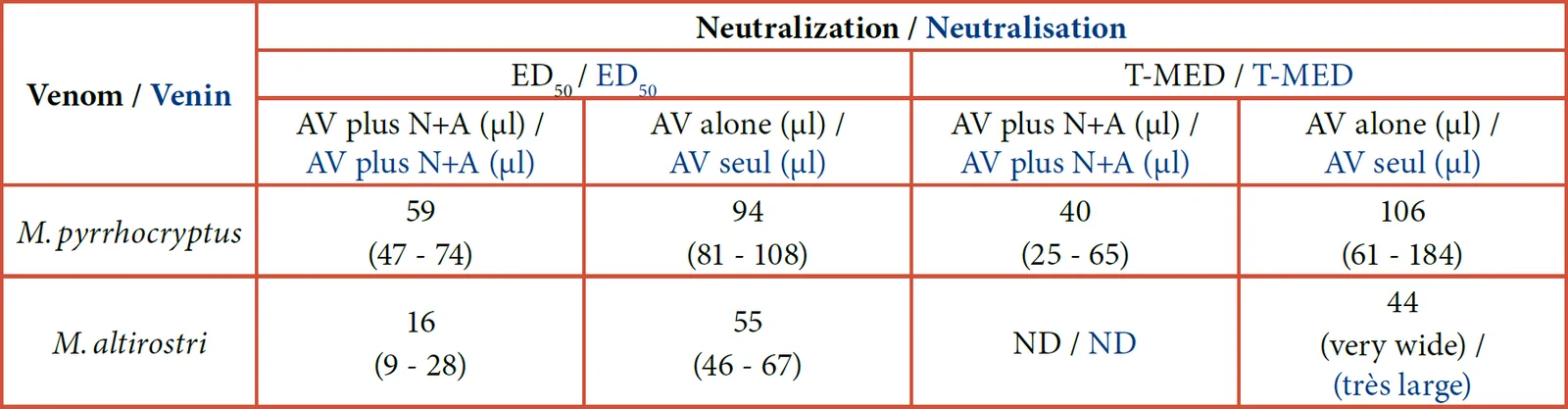
ED50 and T-MED of the AV with or without N+A. In all cases, both the actual values and the theoretical values obtained from the rescue experiments fell within the range of the 95% confidence intervals. ND = not determined

**Appendix VII:**
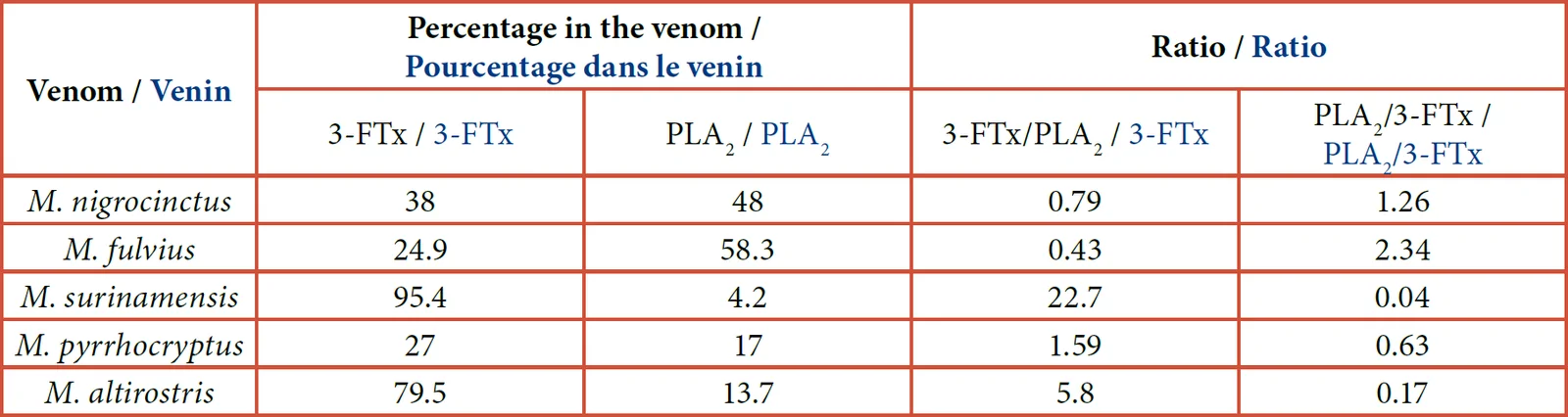
Percentage of α-neurotoxins (3-FTx) and phospholipases A_2_ (PLA2) of the venoms from Micrurus species used in this study, and their relation. Data of percentage were obtained from proteomic studies (see Discussion).
