# Supplementary material for: Usefulness of the combination of a single dose of neostigmine-atropine plus antivenom on the experimental treatment of the envenomation caused by Micrurus venoms
Source: Med Trop Sante Int. 2026 Jul 2;6(3):mtsi.v6i3.2026.871. doi: 10.48327/mtsi.v6i3.2026.871 (PMC13714821; doi:10.48327/mtsi.v6i3.2026.871)
Supplement: Related texts

# Research article: Utilité de l’association d’une seule dose de néostigmine-atropine et d’un antisérum dans le traitement expérimental de l’envenimation causée par les venins du genre *Micrurus*

## Introduction

Les serpents corail (genres *Micrurus*, *Micruroides* et *Leptomicrurus*) constituent le groupe de serpents le plus diversifié d’Amérique. Ce sont les seuls élapidés terrestres d’Amérique et ils peuplent un large éventail d’écosystèmes terrestres — du niveau de la mer jusqu’à environ 3 000 m d’altitude —, allant des régions désertiques froides et chaudes aux zones tropicales humides et chaudes, et s’étendant géographiquement du nord de la Patagonie au Mexique et au sud des États-Unis [13]. Le genre *Micrurus* comprend environ 70 espèces [13,21,58].

Le venin des serpents *Micrurus* provoque une perte de force musculaire et une paralysie respiratoire d’origine périphérique. Ces effets neuro-toxiques sont principalement dus à l’action des α-neurotoxines (dotées d’une structure de toxine à trois doigts (3-FTx)), qui bloquent les récepteurs de l’acétylcholine au niveau de la plaque motrice, dans la jonction neuromusculaire, et/ ou des β-neurotoxines (dotées d’une structure de phospholipase A_2_ (PLA_2_)) qui inhibent la libération d’acétylcholine provoquée par les terminaisons nerveuses motrices au niveau présynaptique. Dans certains cas, ces effets sont encore exacerbés par l’action additive de composants myotoxiques sur les membranes des fibres mus-culaires [4,6,10,37,47,67,68,75]. Parmi les divers composants toxiques des venins de *Micrurus*, les neurotoxines 3-FTx et PLA_2_ sont considérées comme les plus importantes [35,42].

Le traitement spécifique de l’envenimation par *Micrurus* consiste en l’administration d’un antivenin (AV) correspondant. Néanmoins, ces AV sont rares, difficiles à produire et ne sont actuellement fabriqués que par un nombre limité de producteurs [26]. Contrairement à la large réactivité croisée et à la neutralisation souvent observées parmi les AV ciblant les venins de vipéridés en Amérique [5,22,24,61], les AV contre les serpents corail présentent généralement une réactivité croisée limitée [26, 27]. Cela est probablement attribuable aux différences qualitatives et quantitatives dans la composition des venins de *Micrurus* [42].

L’utilisation d’inhibiteurs de l’acétylcholines-térase (IAChE) comme la néostigmine (N) en traitement d’appoint en cas d’envenimation due à des morsures de serpents corail est suggérée par certains ministères de la santé sudamé-ricains [45,46]. Des données expérimentales soutiennent l’utilité potentielle de ce type de composés pour réduire la neurotoxicité induite par le venin de *Micrurus* [12,20,67]. Les IAChE inhibent l’activité de l’acétylcholinestérase, augmentant ainsi les taux d’acétylcholine au niveau de la jonction neuromusculaire, où l’excès d’acétylcholine peut déplacer de manière compétitive les α-neurotoxines des récepteurs nicotiniques [4,37,43]. En cas d’envenimation par des élapidés, ces médicaments sont généralement administrés en association avec de l’atropine (A) afin d’atténuer leurs effets muscariniques [7,37,43]. Bien que les IMACh soient largement utilisés dans le traitement des envenimations par les élapidés asiatiques, dont les venins contiennent princi-palement des α-neurotoxines [1,4,29,33,36,37,4 2,48,56,73,74,76], les données cliniques sur leur efficacité dans les envenimations par *Micrurus* sont peu nombreuses.

Dans les cas d’envenimations enregistrés au Brésil, la réponse au traitement a été jugée favorable lorsque les IMA ont été utilisés en complément d’un traitement antivenimeux [10,11]. Cependant, la réponse clinique à ces médicaments dépend de la présence de neurotoxines α et de l’absence ou de la faible concentration de neurotoxines β. Leur mécanisme d’action, comme mentionné cidessus, se limite à déplacer les neurotoxines α des récepteurs nicotiniques grâce à des concentrations élevées d’acétylcholine au niveau de la plaque motrice [4,37]. Des études *in vitro* ont démontré l’efficacité des IMA contre l’envenimation expérimentale par les venins de *M. pyrrhocryptus*, *M. frontalis* et *M. lemniscatus* [12,67], trois venins contenant des neurotoxines α [15, 51,60]. En revanche, leur efficacité n’a pas été observée contre des venins à plus forte teneur en neurotoxines β, comme celui de *M. corallinus* [19,70]. Audelà des études fondatrices de Vital Brazil, les recherches expérimentales *in vivo* sur l’utilisation des IMA dans l’envenimation par les serpents corail d’Amérique du Sud sont très rares, voire inexistantes [67].

En Argentine, huit espèces de *Micrurus* ont été décrites : *M. pyrrhocryptus, M. altirostris, M. balyocoriphus, M. tricolor, M. frontalis, M. lemniscatus carvalhoi, M. silviae* et *M. corallinus* [26,45]. Parmi cellesci, *M. pyrrhocryptus* présente la plus large distribution et est probablement responsable de la majorité des morsures de serpents corail dans le pays [26]. Son venin est le principal immunogène utilisé dans la production du sérum antivenimeux actuellement employé en Argentine [23,26]. On ne dispose que de données limitées sur les venins des espèces de *Micrurus* décrites en Argentine [12,25,26,27,28,51]; néanmoins, la présence d’α-neurotoxines dans les venins de ces espèces a été décrite [12,19,28,31,51,60]. Cela confirme le rôle potentiel des inhibiteurs de l’AChE (IAChE) en tant que traitements d’appoint dans la prise en charge des envenimations causées par la plupart de ces espèces.

Dans cette étude, nous avons évalué l’efficacité d’une dose unique d’un IAChE, de l’administration d’un AV sudaméricain de *Micrurus* seul, et de l’association IAChE + AV, dans des modèles murins expérimentaux d’envenimation par *Micrurus*. Des expériences de sauvetage (expériences dans lesquelles l’antivenin est administré à un moment déterminé après l’injection d’une dose létale de venin) ont été menées sur des envenimations expérimentales par *M. pyrrhocryptus* (Fig. 1), *M. altirostris* (Fig. 2), *M. surinamensis* (Fig. 3), *M. nigrocinctus* et *M. fulvius*.

**Figure 1:** *Micrurus pyrrhocryptus*, province de Córdoba, Argentine (crédit photo: Adolfo Rafael de Roodt)

**Figure 2:** *Micrurus altirostris,* province de Misiones, Argentine (crédit photo : Adolfo Rafael de Roodt)

**Figure 3:** *Micrurus surinamensis,* Leticia Colombie (crédit photo : Juan Silva Haad)

## 2. Matériel et méthodes

### 2.1 Venins

Le venin de *M. pyrrhocryptus* a été obtenu à partir de spécimens collectés dans diverses régions d’Argentine et aimablement fournis par le Dr Eduardo Gould (*Fundación de Estudios Biológicos*, Buenos Aires, Argentine). Le venin de *M. altirostris* a été donné par le Dr Alejandro Urs Vogt (*Centro Toxicológico de Misiones*, Oberá, Misiones, Argentine). Le venin de *M. surinamensis* colombien était un don du Dr Juan Silva Haad (Leticia, Colombie). Le venin de *M. fulvius* provenant de Floride (États-Unis) a été obtenu auprès du Miami Serpentarium (Floride, États-Unis) et celui de *M. nigrocinctus* provenant du Costa Rica a été acquis auprès du *La Nauyaca Serpentarium* (Cuernavaca, Mexique). Tous les venins ont été obtenus par extraction manuelle, immédiatement séchés sous vide ou lyophilisés, puis conservés à -20 °C jusqu’à leur utilisation.

### 2.2 Animaux

Toutes les expériences ont été menées sur des souris CF-1 de 18 à 22 g provenant de l’animalerie de l’Instituto Nacional de Producción de Biológicos (INPB). Les animaux ont été hébergés dans des conditions environnementales contrôlées et ont reçu de la nourriture pour rongeurs et de l’eau filtrée *ad libitum*. L’autorisation éthique pour la manipulation des animaux a été accordée par l’INPB – ANLIS « Dr Carlos G. Malbrán », conformément aux directives du Conseil national de la recherche [49,50] et de la Fédération des associations européennes des sciences des animaux de laboratoire [30].

### 2.3 Néostigmine (N) et atropine (A)

La néostigmine (Neostigmina Drawer, Laboratory Drawer) a été utilisée en ampoules de 1 ml à une concentration de 0,5 mg/ml. L’atropine (Klonatropina, Klonal Laboratorios) a été utilisée en ampoules de 1 ml à une concentration de 1 mg/ml. Tous les médicaments ont été utilisés avant leur date de péremption.

### 2.4 Antivenin

L’antivenin utilisé était l’*Antiveneno Elapídico*, produit par l’INPB après immunisation de chevaux avec des venins d’espèces argentines du genre *Micrurus*, principalement *M. pyrrhocryptus* [26], lot n° 133, date d’expiration 31 août 2017. L’AV est constitué de fragments F(ab')_2_. Bien que l’efficacité des AV après leur date de péremption ait été documentée [17,52,53,59,63], cet AV a été testé pour ses propriétés physicochimiques et sa capacité neutralisante avant utilisation, confirmant qu’il conservait toutes les caractéristiques déclarées par le fabricant.

### 2.5 Détermination des doses létales médianes, des doses mortelles minimales et du délai avant la mort

La puissance létale des venins a été déterminée par voie souscutanée (s.c.). Des groupes de souris (n = 5 par dose) ont reçu des injections de différentes quantités de venin dilué dans du NaCl 0,15 M, pour un volume final de 0,3 ml. La mortalité a été enregistrée 24 h après l’injection. La puissance létale a été définie comme 1) la dose létale médiane (DL_50_), représentant la dose de venin nécessaire pour tuer 50 % des souris exposées dans un délai de 24 h, et 2) la dose mortelle minimale (DMM), la dose la plus faible de venin ayant entraîné une mortalité de 100 % dans le même délai (équivalente à la DL_100_). Les courbes dosemortalité ont été analysées à l’aide d’un modèle dose-réponse sigmoïdal à pente variable [14].

Une fois la DMM établie, des groupes de souris ont reçu une injection de 1,2 DMM de chacun des venins, et le délai entre l’injection et la mort a été enregistré. Les comparaisons entre groupes ont été effectuées à l’aide du test de Mann-Whitney ou du test *t* de Student, selon le cas.

### 2.6 Dosages de la néostigmine et de l’atropine

Plusieurs doses de N et d’A ont été évaluées sur la base des plages de doses précédemment utilisées dans des modèles murins d’intoxication et d’envenimation impliquant une inhibition de l’AChE [18,34,38,41]. Les doses finales retenues étaient de 0,25 μg/g de N et de 2,0 μg/g d’A. Les deux médicaments ont été administrés par voie intrapéritonéale (i.p.) dans un volume final de 0,5 ml/souris, dilués dans une solution de NaCl 0,15 M. En association avec l’AV, le même volume final et le même diluant ont été utilisés, avec des ajustements appropriés pour le volume d’AV. Les doses d’A et de N ont été testées seules et en association, jusqu’à trois fois supérieures à la dose expérimentale finale, sur des groupes de cinq souris afin de s’assurer de l’absence de toxicité. Aucun effet indésirable n’a été observé à ces niveaux. Des signes graves d’intoxication et de mortalité (2 animaux sur 5 exposés) n’ont été observés qu’à des doses cinq fois supérieures à celles sélectionnées.

### 2.7 Expériences de neutralisation

**2.7.1 Effet de différentes doses d’AV avec ou sans N+A**

**2.7.1.1 Groupes expérimentaux**

Des groupes de cinq souris ont reçu une injection sous-cutanée de 1,2 DMM de chaque venin, dilué dans une solution de NaCl 0,15 M pour un volume final de 0,3 ml. Quinze minutes après l’envenimation, les souris ont reçu un traitement de secours intrapéritonéal (i.p.) consistant en AV seul (50 ou 100 μl) ou en AV associé à N+A, dilué dans 0,15 M de NaCl pour un volume final de 0,5 ml. Les groupes de traitement étaient les suivants: groupe 1) N (5 μg) + A (40 μg); groupe 2) différentes doses d’AV (50 ou 100 μl) plus N (5 μg) + A (40 μg); groupe 3) AV seul (50 ou 100 μl); groupe 4) venin seul dilué dans du NaCl 0,15 M (témoin positif).

**2.7.1.2 Interprétation des données**

La protection conférée par chaque traitement a été exprimée en pourcentage de souris survivantes 24 h après l’injection. Les données de survie (nombre de décès et de survivants par groupe) ont été analysées à l’aide du test exact de Fisher afin de comparer l’efficacité de l’AV seul à celle de l’AV associé à N+A.

**2.7.1.3 Retard relatif de la mort avec N+A seul**

En raison des délais de mort assez différents observés après l’administration de 1,2 DMM des différents venins, le délai de mort a été enregistré pour chaque animal. Afin d’évaluer le bénéfice potentiel du traitement par N+A pour retarder ou prévenir la mort pour chaque venin testé, le délai relatif avant la mort (DRM) a été défini comme le rapport entre le délai moyen avant la mort chez les souris traitées par N+A et le délai moyen avant la mort chez les témoins (venin seul), selon la formule : DRM = (délai avant la mort avec N+A) / (délai avant la mort chez les témoins).

**2.7.1.4 Analyse des données de survie**

Les résultats de survie médiane issus des différentes expériences ont été analysés à l’aide de modèles de régression linéaires et non linéaires. Les valeurs médianes dérivées des analyses doseréponse sigmoïdales ont été considérées comme des doses médianes théoriques (T-MED). De plus, les rapports entre les valeurs T-MED obtenues avec et sans traitement par N+A ont été calculés et analysés.

**2.7.2 Effet du N+A sur la dose efficace médiane de sauvetage de l’AV contre les venins de** ***M. pyrrhocryptus*** **et** ***M. altirostris***

Afin d’évaluer si l’utilisation du N+A modifie la dose efficace médiane (DE_50_) de l’AV contre l’envenimation par les venins de *M. pyrrhocryptus* et *M. altirostris*, les valeurs de sauvetage DE_50_ (r-DE_50_) ont été estimées dans des tests DE_50_ avec et sans co-administration de N+A. Des groupes de 5 souris ont reçu une injection sous-cutanée dans la région dorsale de 1,2 DMM de venin dans un volume final de 0,3 ml de NaCl 0,15 M. Au bout de quinze minutes, les souris ont reçu une injection intraperitoneale de différentes doses d’AV seul ou d’AV associé à N+A, dans un volume final de 0,5 ml/souris, dans une solution de NaCl 0,15 M. Les souris ont été surveillées et les décès ont été enregistrés après 24 heures. La dose efficace médiane de sauvetage (r-DE_50_) a été définie comme la dose d’AV protégeant 50 % des souris [54] et a été estimée à l’aide d’un modèle dose-réponse sigmoïdal à pente variable [14].

### 2.8 Statistiques

La distribution des données a été évaluée à l’aide du test de Kolmogorov-Smirnov. Pour la compa-raison des groupes, le test de Mann-Whitney ou le test t de Student ont été utilisés. Pour l’analyse des comparaisons multiples, des tests ANOVA ou de Kruskal-Wallis avec ajustement de Bonferroni ou de Dunn ont été utilisés. Les différences de fréquences ont été analysées à l’aide du test exact de Fisher. Les expériences dose-réponse ont été analysées par régression linéaire ou non linéaire à l’aide d’un modèle dose-réponse sigmoïdal à pente variable. Toutes les analyses statistiques ont été réalisées à l’aide du logiciel Prism 6.0 (GraphPad Inc., CA, États-Unis) pour Mac. Une valeur de p ≤ 0,050 a été considérée comme statistiquement significative.

## 3 Résultats

### 3.1 Puissances létales

Les DL_50_ et DL_100_ (DMM) par voie souscutanée des venins utilisés dans cette étude étaient de 31 μg et 45 μg pour *M. pyrrhocryptus*, 26 μg et 30 μg pour *M. altirostris*, 28 μg et 40 μg pour *M. surinamensis*, 27 μg et 57 μg pour *M. nigrocinctus* et 22 μg et 40 μg pour les venins de *M. fulvius*. Ces doses et les masses de venin correspondant aux doses d’essai de 1,2 DMM utilisées dans cette étude sont détaillées dans le Tableau I.

**Tableau I:** Dose létale médiane (DL_50_), dose mortelle minimale (DMM ou DL_100_) et dose de provocation (1,2 DMM) des venins de Micrurus utilisés dans cette étude, exprimées en microgrammes par souris de 20 g

Venom	Toxic Doses		
	LD_50_ s.c. (μg/mouse)	LD_100_ s.c. (MMD) (μg/mouse)	Challenge dose (1.2 MMD) (μg/mouse)
*M. pyrrhocryptus*	31 (28 to 35)*	45	54
*M. altirostris*	26 (25-27)*	30	36
*M. surinamensis*	28 (25 to 30)*	40	48
*M. nigrocinctus*	27 (21 to 35)*	57	68
*M. fulvius*	22 (19 to 27)*	40	48

* values between prentheses are the 95% confidence intervals

### 3.2 Délai avant la mort des souris injectées de venin avec ou sans traitement par N+A

Le venin de *M. surinamensis* a provoqué la létalité la plus rapide, avec un délai moyen avant la mort de 25 ± 6 min, tandis que le venin de *M. fulvius* a entraîné la progression la plus lente vers la mort, avec un délai moyen de 157 ± 35 min (ANOVA-Bonferroni p < 0,025 et p < 0,0001 pour *M. surinamensis* et *M. fulvius* respectivement). Le délai moyen avant la mort pour les autres venins était de 43 ± 6 min, 47 ± 11 min et 54 ± 9 min pour les venins de *M. nigrocinctus*, *M. pyrrhocryptus* et *M. altirostris* respectivement (Tableau II). Le DRM est représentée sur la Figure 4.

**Tableau II:** Délai avant la mort provoquée par l'injection de 1,2 DMM des différents venins, avec et sans injection de N+A

Venom	Treatment	Time of death (min)				n	p
		Mean ± SD	Median	Minimum	Maximum		Statistic
*M. pyrrhocryptus*	Venom alone	47 ± 11	48	30	75	22	< 0.05 (U = 174,5; t = 2.09)*
	Venom plus N+A	55 ± 15	50	45	114	24	
*M. altirostris*	Venom alone	54 ± 9	54	41	70	17	< 0.001 (U = 20.5)
	Venom plus N+A	76 ± 14	78	51	101	15	
*M. surinamensis*	Venom alone	25 ± 6	24	18	42	18	< 0.001 (U = 0.0)
	Venom plus N+A	60 ± 27	53	43	122	8	
*M. nigrocinctus*	Venom alone	43 ± 6	42	34	56	13	0.69 (U = 76.5)
	Venom plus N+A	43 ± 4	42	36	51	13	
*M. fulvius*	Venom alone	157 ± 35	151	110	217	12	0.29 (U = 53.5)
	Venom plus N+A	132 ± 46	147	59	190	12	

n : nombre d'animaux; U : valeur (test de Mann-Whitney); * : test t de Student); dans ce cas (venin de *M. pyrrhocryptus*), les différences entre les groupes ont été analysées à l'aide des méthodes du test t de Student et du test de Mann-Whitney

**Figure 4:** Délai relatif avant la mort (DRM) après traitement par N+A. Les barres représentent le rapport entre le temps jusqu’au décès des souris ayant reçu une injection de V et un traitement par N+A, et le temps jusqu’au décès des souris témoins ayant reçu uniquement une injection de V (DRM = V + N+A/V seul)

L’association N+A n’a pas protégé les souris contre la dose de provocation, mais a retardé le délai de mort pour les venins de *M.surinamensis, M.altirostris* et *M. pyrrhocryptus*. Ce n’est que dans le cas de *M.altirostris* que la survie de 2 souris sur 15 traitées par le venin et N+A a été observée (Tableau II).

### 3.3 Survie des souris injectées de venin et sauvées par l’AV seule ou par l’AV associée à N+A

L’association du traitement par l’AV et N+A a permis d’obtenir de meilleurs résultats dans les cas d’envenimations par des espèces sudaméri-caines de *Micrurus*.

**3.3.1 Venin de** ***M. pyrrhocryptus***

L’ajout de N+A à l’AV a donné de meilleurs résultats que le traitement par l’AV seule (Tableau III; Annexe 2). Parmi les souris exposées à ce venin, le taux de survie était plus élevé dans le groupe traité avec 100 μl d’AV plus N+A (21 souris sur 25 ont survécu avec l’ajout de N+A, contre 11 sur 25 dans le groupe AV seul) ou avec 50 μl d’AV (13 survivants sur 25 avec N+A contre 7 sur 25 avec l’AV seul). Le test exact de Fisher a confirmé une amélioration statistiquement significative avec 100 μl d’AV plus N+A par rapport à 100 μl d’AV seul (p = 0,0072) (Fig. 5; Fig. 6; Annexe 2). Bien que la protection semblait plus élevée avec 50 μl d’AV plus N+A par rapport à 50 μl d’AV seul, dans ce test, la différence n’a pas atteint le seuil de signification statistique (p = 0,0833). Néanmoins, des différences statistiques ont été observées lorsque la protection fournie par cette dose d’AV plus N+A par rapport à l’AV seul dans les expériences DE_50_ (n : 30 souris) a été analysée (p= 0,0352) (Annexe 3.a). Cette différence a été observée lorsque toutes les données utilisant une dose de 50 μl d’AV seul ou associé à N+A (doses fixes + essais DE_50_, n: 80 souris) ont été analysées (p = 0,0032) (Annexe 3.b).

**Figure 5:** Réponse générale aux différents traitements. Résultats de survie et de mortalité chez des souris exposées à 1,2 DMM de venin et traitées soit par AV seul, soit par AV associé à N et A (des doses allant jusqu’à 250 μL ont été incluses). Les barres représentent les résultats cumulés de toutes les expériences. Les parties noires indiquent les souris décédées, et les parties blanches indiquent les souris survivantes. Les astérisques indiquent des différences statistiquement significatives (p < 0,05, test exact de Fisher) entre les taux de survie et de mortalité des souris traitées par AV seul et celles recevant AV plus N+A

**Figure 6:** Réponse à différentes doses d’AV avec ou sans N+A. Les résultats de survie et de mortalité chez les souris exposées à 1,2 DMM de venin (V) et traitées avec différentes doses d’AV seul ou d’AV associé à N+A ont été comparés à l’aide du test exact de Fisher. Les barres représentent les données cumulées par dose. Les parties noires indiquent les souris décédées, et les parties blanches indiquent les souris survivantes. Les astérisques indiquent des différences statistiquement significatives (p < 0,05).

**Tableau III:** Pourcentage de survie des souris exposées à 1,2 DMM de venin et sauvées par différentes doses fixes d’AV seul ou en association avec N+A

Venom	Treatment	Dose AV (μL)	Protection (%)			Number of experiments
			Median	Minimum	Maximum	
*M. pyrrhocryptus*	AV alone / AV seul	50	20	0	60	5
		100	40	20	60	5
		250	100	*	*	1
	AV plus N+A / AV plus N+A	50	60	0	100	5
		100	80	60	100	5
		250	100	*	*	1
*M. altirostris*	AV alone / AV seul	50	60	0	60	5
		100	60	40	80	5
		250	100	*	*	1
	AV plus N+A / AV plus N+A	50	100	60	100	5
		100	100	80	100	5
		250	100	*	*	1
*M. surinamensis*	AV alone / AV seul	50	0	0	0	2
		100	0	0	0	2
		250	20	0	20	3
	AV plus N+A / AV plus N+A	50	0	0	0	2
		100	20	0	80	5
		250	60	0	100	5
*M. nigrocinctus*	AV alone / AV seul	50	0	0	0	3
		100	0	0	0	4
		250	40	*	*	1
	AV plus N+A / AV plus N+A	50	0	0	0	3
		100	10	0	20	4
		250	40	*	*	1
*M. fulvius*	AV alone / AV seul	50	10	0	20	2
		100	0	0	0	3
		200	40	*	*	1
		400	60	*	*	1
	AV plus N+A / AV plus N+A	50	10	0	20	2
		100	20	0	20	3
		200	40	*	*	1
		400	100	*	*	1

Cinq souris ont été utilisées par expérience.

**3.3.2 Venin de** ***M. altirostris***

L’ajout de N+A au traitement par AV a augmenté la survie. Parmi les souris exposées, l’utilisation de la combinaison a donné de meilleurs résultats (Tableau III; Annexe 2) : avec une dose de 100 μl d’AV, 16 souris sur 25 ont survécu dans le groupe AV seul, contre 23 sur 25 dans le groupe AV plus N+A. Lorsque la dose d’AV était de 50 μl, la combinaison AV plus N+A a protégé 22 sur 25, tandis que l’utilisation de l’AV seul n’a protégé que 12 souris sur 25. Le test exact de Fisher a montré que l’association de 100 ou 50 μl d’AV avec N+A offrait une protection significativement meilleure que l’AV seul (p = 0,0374 et 0,0054, respectivement) (Fig. 5; Fig. 6; Annexe 2).

**3.3.3 Venin de** ***M. surinamensis***

Dans l’ensemble, l’AV seul n’a protégé qu’une seule souris sur 15 à une dose de 250 μl (1/15), tandis que 100 ou 250 μl d’AV associés à N+A ont protégé respectivement 8/25 et 15/25 souris. Aucune protection n’a été observée avec 50 μl d’AV associés à N+A (0/10) (Tableau III). Le test exact de Fisher a montré que le meilleur résultat a été obtenu avec 250 μL d’AV plus N+A (p = 0,0009). L’utilisation de 100 μl d’AV et de N+A, bien qu’apparemment plus efficace, ne différait pas significativement de l’AV seul (p = 0,0734) (Fig. 5; Fig. 6; Annexe 2).

**3.3.4 Venin de** ***M. nigrocinctus***

Aucune différence significative en termes de survie n’a été observée entre l’AV seul et l’AV combiné à N+A; 2/40 contre 5/40 souris ont survécu (n = 80) (Tableau III; Fig. 6). Lorsque chaque dose a été analysée séparément à l’aide du test exact de Fisher, aucune différence statistiquement significative n’a été constatée (p > 0,4 dans tous les cas) (Fig. 5; Fig. 6; Annexe 2).

**3.3.5 Venin de** ***M. fulvius***

Aucune différence statistiquement significative n’a été observée en termes de survie entre le traitement par AV seul et celui par AV associé à N+A. En examinant les différentes doses, un certain degré de protection a été observé (Tableau III), mais le test exact de Fisher n’a révélé aucune différence significative (p > 0,4 dans tous les cas) (Fig. 5; Fig. 6; Annexe 2).

### 3.4 Variation de la dose médiane efficace de sauvetage de l’AV avec ou sans N+A

L’ajout de N+A au traitement par AV a renforcé son pouvoir neutralisant contre les deux venins, bien que l’ampleur de l’amélioration ait varié. Pour le venin de *M. altirostris*, la DE_50_ de l’AV seul était de 55 μl (IC à 95 % : 46–67 μl, R^2^ = 0,9781), valeur qui est tombée à 16 μl (9–28 μl, R^2^ = 0,7655) lorsqu’il était associé à N+A, ce qui reflète une multiplication par 3,4 du pouvoir neutralisant. En revanche, pour le venin de *M. pyrrhocryptus*, la DE_50_ de l’AV seule était de 94 μl (81–108 μl, R^2^ = 0,7727), et celleci a été réduite à 59 μl (47–74 μl, R^2^ = 0,8273) avec l’ajout de N+A, ce qui correspond à une amélioration de 1,6 fois (Fig. 7).

**Figure 7:** Dose efficace médiane d’AV, administrée seule ou en association avec N+A, dans les expériences de sauvetage. L’AV ou l’AV plus N+A a été injecté par voie intrapéritonéale 15 minutes après l’injection sous-cutanée de 1,2 dose minimale mortelle (MMD) des venins respectifs. La ligne continue représente la réponse à l’AV seul, tandis que la ligne pointillée représente la réponse à l’AV plus N+A. Dans les deux cas, l’ajout de N+A a réduit la dose d’AV nécessaire pour sauver 50 % des souris exposées. A. Contre le venin de *M. pyrrhocryptus,* la DE_50_ de l’AV seul était de 94 μl (IC à 95 % : 81 – 108 μl, R^2^ = 0,7727), tandis que l’ajout de N+A a réduit la DE_50_ à 59 μl (47–74 μl, R^2^ = 0,8273), multipliant ainsi par 1,6 le pouvoir neutralisant. B. Pour le venin de *M. altirostris*, la DE_50_ de l’AV seule était de 55 μl (46–67 μl, R^2^ = 0,9781), réduite à 16 μl (9–28 μl, R^2^ = 0,7655) lorsqu’elle était combinée avec N+A, ce qui correspond à une augmentation de 3,4 fois de la puissance neutralisante

### 3.5 Analyse des doses utilisées et de la protection

Dans tous les cas, le traitement a amélioré l’issue des envenimations expérimentales à des degrés divers. Cependant, des différences statistiquement significatives n’ont été observées que pour les venins de *M. altirostris* et de *M. pyrrhocryptus*, ainsi que pour le venin de *M. surinamensis*, mais

uniquement avec des doses élevées d’AV (Fig. 5 et Fig. 6). Une analyse préliminaire utilisant une régression linéaire et non linéaire des valeurs médianes de protection en fonction des doses d’AV a confirmé cette tendance (Annexes 4 et 5). Les données indiquent que l’ajout de N+A permet de réduire la dose d’AV nécessaire pour obtenir le même niveau de protection pour tous les venins testés (Tableau III; Fig. 5 et Fig. 8; Annexe 4 et Annexe 5).

**Figure 8:** Les valeurs de survie médiane issues des différentes expériences de sauvetage sont représentées sous forme de pourcentages en fonction des doses d’AV correspondantes. Les traitements utilisant l’AV seul sont représentés par des losanges noirs: ♦ et les traitements utilisant l’AV plus N+A par des cercles blancs: O. Les étoiles (*) indiquent les points de données pour lesquels une seule expérience a été réalisée (doses les plus élevées).

En ce qui concerne la neutralisation des venins de *M. pyrrhocryptus* et de *M. altirostris* en particulier, bien que les valeurs de la dose efficace (DE_50_) et de la dose médiane (T-MED) différaient de près du double, leurs intervalles de confiance à 95 % se chevauchaient (Fig. 7; Annexe 6).

## 4. Discussion

L’envenimation par les serpents corail est rare, mais lorsqu’elle survient, elle constitue une urgence médicale pour laquelle le seul traitement efficace est l’administration d’un antivenin spécifique [45,46]. Cet antivenin est difficile à produire et sa disponibilité est limitée, même dans les pays disposant de capacités de fabrication locales [10,11,23,25,27].

Les inhibiteurs de l’AChE, seuls ou associés à des antivenins, ont été utilisés pour le traitement de l’envenimation par d’autres élapidés [4,37,48]. Bien que certains bénéfices expérimentaux aient été observés dans les cas d’envenimation causée par des venins contenant à la fois des neuro-toxines présynaptiques et postsynaptiques [76], l’expérience clinique montre une absence ou de faibles bénéfices dans les cas d’envenimation par d’autres élapidés. Dans les cas d’envenimation causée par des venins contenant des neurotoxines présynaptiques, comme les espèces des genres *Bungarus* ou *Oxyuranus* [2,62,65,72], les bénéfices sont moindres ou inexistants par rapport à leur utilisation dans les envenimations par des venins contenant principalement des neurotoxines post-synaptiques comme *Naja* [29,36,56,73,74,76]. Dans le cas des serpents corail, l’utilisation d’inhibiteurs de l’AChE est particulièrement recommandée en cas d’envenimation par des serpents dont le venin se compose exclusivement de neurotoxines postsynaptiques [9,10,46,67].

Comme mentionné précédemment, l’utilisation d’inhibiteurs de l’AChE a été proposée comme approche thérapeutique pour les envenimations par *Micrurus* [32,67,69,70,71]; néanmoins, certains venins de *Micrurus*, tels que ceux de *M. corallinus* et de *M. fulvius,* provoquent également une neurotoxicité présynaptique via des β-neurotoxines [19,66,67]. L’action de ces β-neurotoxines, ainsi que de certaines PLA_2_ non neurotoxiques, implique des mécanismes supplémentaires pouvant causer des lésions au niveau des membranes cellulaires, ce qui peut compliquer l’évolution de l’envenimation [4,6,47]. En raison de cette variabilité importante, les inhibiteurs de l’AChE ne sont pas toujours efficaces et leur rôle pourrait être plus approprié en tant que traitement d’appoint plutôt que comme modalité thérapeutique principale [8,10,11].

Comme mentionné cidessus, l’issue chez les souris traitées est liée à la composition des venins, en particulier au type de neurotoxines. On aurait pu s’attendre à ce que l’ajout de N+A donne de bons résultats avec des venins présentant des proportions plus élevées d’α-neurotoxines, tels que *M. surinamensis* (95,4 %) [60] et *M. altirostris* (79,5 %) [19]. Dans le cas de *M. altirostris*, les bénéfices étaient évidents, mais dans le cas de *M. surinamensis*, les résultats pourraient être liés aux caractéristiques particulières de ce venin [55,64], à la non-spécificité de l’antivenin telle qu’observée dans des études précédentes [26, 27] et à la difficulté à neutraliser ce venin, même avec un antivenin spécifique [26]. Néanmoins, l’utilisation de N+A à fortes doses améliore l’efficacité de l’AV contre ce venin.

Cependant, malgré la présence de 3-FTx dans le venin de *M. nigrocinctus* (38 %) [31] et dans celui de *M. fulvius* (24,9 %) [44,66], l’amélioration a été inférieure à celle observée avec le venin de *M. pyrrhocryptus*, avec 27 %. L’efficacité limitée ou absente de l’association AV plus N+A contre les envenimations par *M. fulvius* et *M. nigrocinctus* aux doses testées peut être due à la présence de PLA_2_ myotoxiques et neurotoxiques chez *M. fulvius* [44,66], représentant plus de 58 % de la masse du venin, et de PLA_2_ myotoxiques représentant 48 % de la masse du venin chez *M. nigrocinctus* [25,35,44,57,66] (Annexe 7).

Dans les envenimations expérimentales utilisant le venin de *M. fulvius*, une bonne neutralisation a été observée à l’aide d’inhibiteurs de la PLA_2_ [39,40], ce qui renforce le rôle important de la β-neurotoxicité dans cette envenimation. En revanche, malgré la forte concentration en PLA_2_ dans le venin de *M. nigrocinctus*, sa β-neurotoxicité ne semble pas jouer un rôle significatif dans l’envenimation [31], contrairement à sa myotoxicité [25, 31, 57]. Le venin de *M. pyrrhocryptus* présente une faible myotoxicité expérimentale [12,25], activité non observée dans les venins de *M. surinamensis* et *M. altirostris* [25]. Étant donné que les N+A ne neutralisent pas ces mécanismes toxiques, cela pourrait expliquer les résultats obtenus dans ces envenimations dans la présente étude.

Au Brésil, l’utilisation d’inhibiteurs de l’AChE est recommandée comme intervention d’appoint lorsque l’administration d’antidotes antivenimeux (AV) n’est pas disponible, retardée ou insuffisante, ou chez les patients qui ne présentent pas d’amélioration après avoir reçu les doses maximales recommandées d’AV, ou qui présentent un rétablissement retardé, voire des manifestations neurotoxiques systémiques récurrentes [9,10]. Des études *in vitro* ont montré que le blocage neuromusculaire induit par diverses espèces de *Micrurus*, par exemple *M. altirostris, M. laticollaris, M. nigrocinctus, M. pyrrhocryptus* et *M. spixii*, implique des interactions avec les récepteurs nicotiniques postsynaptiques. Cependant, la réversion induite par la N est généralement transitoire et souvent minime ou discrète (généralement <30 %), n’offrant aucune protection substantielle à long terme contre le blocage neuromusculaire progressif. Une réversion complète de la neurotoxicité postsynaptique n’a été obtenue qu’avec l’administration d’AV [9, 10,11]. Magalhães Camargo *et al.* ont démontré que le traitement par N inversait de manière transitoire le blocage neuromusculaire après une inhibition de 50 % induite par le venin de *M. pyrrhocryptus*, mais qu’un blocage complet et irréversible survenait après 60 minutes. Cela pourrait expliquer la protection limitée offerte par une dose unique de N+A et l’amélioration de la survie observée lors de l’association de N+A avec l’AV [1,4, 33,38].

Dans les cas d’envenimation par des serpents, il est très important d’instaurer rapidement le traitement spécifique, mais cela est particulièrement important dans le cas des envenimations neurotoxiques, car l’efficacité optimale est obtenue lorsque l’AV empêche la liaison des toxines à leurs cibles [3,16]. Il en va de même pour l’utilisation de

N, qui donnerait de meilleurs résultats au début de l’envenimation. Au Brésil, de bons résultats ont été observés dans les cas d’envenimation par *Micururus* grâce à l’association d’AV et de N+A, lorsqu’elle était administrée 2 heures et 5 heures après la morsure, mais en utilisant deux doses de N+A [11]. Néanmoins, les efforts doivent être orientés vers l’utilisation précoce du N+A. Malheureusement, les études dose-réponse menées pour comparer les DE_50_ des AV seules ou combinées avec N+A n’ont pu être réalisées que pour les venins de *M. pyrrhocryptus* et *M. altirostris*. Nous ne disposions pas de suffisamment de matériel pour déterminer les DE_50_ de tous les venins dans le cadre d’expériences dédiées. Nous espérons obtenir à l’avenir suffisamment de venin et d’AV pour mener d’autres études.

## 5 Conclusions

Dans cette étude, nous avons observé de meilleurs résultats lors de l’association de N+A et d’AV, l’AV utilisé n’étant pas spécifique à l’espèce pour 4 des 5 venins étudiés. Pour les trois venins de serpents corail d’Amérique du Sud testés, cette association a permis d’utiliser des doses d’AV plus faibles par rapport à l’AV seul. Dans ces scénarios, l’association peut s’avérer utile lorsqu’il n’est pas possible d’augmenter la dose d’AV ou lorsque d’autres mesures complémentaires, telles que l’assistance respiratoire [11], ne sont pas disponibles.

Nous n’avons pas observé de résultats positifs dans les cas d’envenimations par *M. nigrocinctus* et *M. fulvius*. Il convient toutefois de noter que l’AV utilisé n’était ni spécifique ni étroitement apparenté à ces venins, et des études antérieures ont montré une faible protection avec cet AV contre ces venins [26,27]. Des études supplémentaires sur l’association AV plus N+A (ou d’autres IMAH) utilisant des AV spécifiques ou plus étroitement apparentés, ou des doses plus élevées d’AV paraspécifiques, pourraient fournir des résultats plus concluants sur le bénéfice potentiel de l’utilisation des IMAH dans ces envenimations. L’association d’AChEI avec des antivenins, et éventuellement avec des inhibiteurs de la PLA_2_, devrait être davantage explorée en tant que stratégie visant à améliorer les traitements existants ou à offrir des options thérapeutiques alternatives pour ces envenimations rares mais très graves, en particulier dans les régions où les antivenins peuvent être rares ou indisponibles.

Nos résultats concordent avec plusieurs observations sur l’utilité de l’association N+A dans certaines envenimations par des Elapidae et, bien qu’expérimentaux, montrent l’aide potentielle de l’association N+A plus antivenin dans ce type d’envenimations. Bien que notre étude, réalisée chez la souris, ne puisse pas entièrement être extrapolée à un usage thérapeutique chez l’homme, les résultats fournissent des informations importantes sur l’utilisation de la combinaison N+A plus antivenin dans les envenimations par des espèces de *Micrurus* dont les venins présentent une composition différente.

À notre connaissance, il s’agit de la première étude *in vivo* sur l’utilité de l’association d’un antivenin anti-*Micrurus* avec des inhibiteurs de l’AChE dans le traitement de l’envenimation par les serpents corail les plus méridionaux d’Amérique, *M. pyrrhocryptus et M. altirostris*.

## Financement

Cette recherche n’a bénéficié d’aucune subvention spécifique de la part d’un organisme de financement, qu’il soit public, privé ou à but non lucratif.

## Contribution des auteurs et autrices

ARdR a conçu l’étude; DD, FAMG, MAD et ArdR ont mené les travaux sur le terrain; ARdR a réalisé l’analyse des données; CFD et ARdR ont supervisé l’étude; ArdR et DD ont relu et révisé la version finale. Tous les auteurs et autrices ont lu et approuvé le manuscrit final.

## Déclaration de liens d’intérêt

Aucun lien d’intérêt n’a été déclaré.

## Annexes

**Annexe I:** Taux de survie (en pourcentage) de souris ayant reçu une dose de 1,2 DMM de différents venins de *Micrurus* et traitées soit par AV seul, soit par une association d’AV et de N+A A : venin de *M. pyrrhocryptus* B : venin de *M. altirostris* C : venin de *M. surinamensis* D : venin de *M. nigrocinctus* E : venin de *M. fulvius* Les barres indiquent la survie médiane pour chaque groupe d’expériences. Chaque point représente une expérience.

**Annexe II:** Test exact de Fisher sur la survie de souris exposées à différents venins et traitées avec différentes doses d’AV, injectées seules ou en association avec N+A (néostigmine et atropine)

**Annexe III:** A. Taux de survie des souris exposées au venin de *M. pyrrhocryptus* et traitées avec 50 μl d’AV seul ou en association avec N+A. Données issues des tests de DE_50_. B. Survie des souris exposées au venin de *M. pyrrhocryptus* et traitées avec 50 μl d’AV seul ou avec l’ajout de N+A. Données issues des tests de DE_50_ (n = 30) et de l’exposition à des doses fixes (n = 50)

**Annexe IVa:** : Régression linéaire de la relation entre les doses d’AV transformées en logarithmes (en μl) et la survie médiane (%) des souris exposées. Les lignes pleines représentent la réponse à l’AV seul, tandis que les lignes pointillées représentent la réponse à l’AV associé à N+A. Dans tous les cas, l’association de l’AV et de N+A a nécessité des doses d’AV plus faibles que celles requises par l’AV seul pour produire le même effet.

**Annexe IVb:** Valeurs de régression et valeurs p pour les résultats présentés au point 4.a. Dans le cas des venins de *M. altirostris* et de *M. surinamensis*, les faibles valeurs de R2 et p s’expliquent par la réponse élevée (pour *M. altirostris*) ou faible (pour *M. surinamensis*) observée aux doses d’AV utilisées.

**Annexe V:** Régression non linéaire de la relation entre les doses d’AV transformées en logarithmes (en μl) et la survie médiane (%) des souris exposées. Les lignes pleines représentent la réponse à l’AV seule, tandis que les lignes pointillées représentent la réponse à l’AV associée à N+A. Dans tous les cas, des doses d’AV plus faibles ont été nécessaires pour obtenir le même effet (Tableau III).

**Annexe VI:** DE50 et T-MED de l’AV, avec ou sans N+A. Dans tous les cas, tant les valeurs réelles que les valeurs théoriques obtenues à partir des expériences de sauvetage se situaient dans l’intervalle de confiance à 95 %. ND = non déterminé

**Annexe VII:** Pourcentage d’α-neurotoxines (3-FTx) et de phospholipases A_2_ (PLA_2_) dans les venins des espèces du genre *Micrurus* utilisées dans cette étude, et leur relation. Les données relatives aux pourcentages ont été obtenues à partir d’études protéomiques (voir Discussion)
